# A New Age in Molecular Diagnostics for Invasive Fungal Disease: Are We Ready?

**DOI:** 10.3389/fmicb.2019.02903

**Published:** 2020-01-14

**Authors:** Sarah E. Kidd, Sharon C.-A. Chen, Wieland Meyer, Catriona L. Halliday

**Affiliations:** ^1^National Mycology Reference Centre, Microbiology and Infectious Diseases, South Australia Pathology, Adelaide, SA, Australia; ^2^Centre for Infectious Diseases and Microbiology Laboratory Services, ICPMR, New South Wales Health Pathology, Westmead Hospital, Westmead, NSW, Australia; ^3^Marie Bashir Institute for Infectious Diseases and Biosecurity, The University of Sydney, Sydney, NSW, Australia; ^4^Molecular Mycology Research Laboratory, Centre for Infectious Diseases and Microbiology, Faculty of Medicine and Health, Westmead Clinical School, The University of Sydney, Sydney, NSW, Australia; ^5^The Westmead Institute for Medical Research, Westmead, NSW, Australia; ^6^Research and Education Network, Westmead Hospital, Westmead, NSW, Australia

**Keywords:** *Aspergillus* PCR, *Candida* PCR, non-culture diagnostics, whole genome sequencing antifungal resistance, *FKS*, *CYP51A*, *ERG11*

## Abstract

Invasive fungal diseases (IFDs) present an increasing global burden in immunocompromised and other seriously ill populations, including those caused by pathogens which are inherently resistant or less susceptible to antifungal drugs. Early diagnosis encompassing accurate detection and identification of the causative agent and of antifungal resistance is critical for optimum patient outcomes. Many molecular-based diagnostic approaches have good clinical utility although interpretation of results should be according to clinical context. Where an IFD is in the differential diagnosis, panfungal PCR assays allow the rapid detection/identification of fungal species directly from clinical specimens with good specificity; sensitivity is also high when hyphae are seen in the specimen including in paraffin-embedded tissue. *Aspergillus* PCR assays on blood fractions have good utility in the screening of high risk hematology patients with high negative predictive value (NPV) and positive predictive value (PPV) of 94 and 70%, respectively, when two positive PCR results are obtained. The standardization, and commercialization of *Aspergillus* PCR assays has now enabled direct comparison of results between laboratories with commercial assays also offering the simultaneous detection of common azole resistance mutations. *Candida* PCR assays are not as well standardized with the only FDA-approved commercial system (T2Candida) detecting only the five most common species; while the T2Candida outperforms blood culture in patients with candidemia, its role in routine *Candida* diagnostics is not well defined. There is growing use of Mucorales-specific PCR assays to detect selected genera in blood fractions. Quantitative real-time *Pneumocystis jirovecii* PCRs have replaced microscopy and immunofluorescent stains in many diagnostic laboratories although distinguishing infection may be problematic in non-HIV-infected patients. For species identification of isolates, DNA barcoding with dual loci (ITS and *TEF1*α) offer optimal accuracy while next generation sequencing (NGS) technologies offer highly discriminatory analysis of genetic diversity including for outbreak investigation and for drug resistance characterization. Advances in molecular technologies will further enhance routine fungal diagnostics.

## Introduction

Invasive fungal diseases (IFDs) pose a significant threat to human health, particularly in the immunocompromised, with an increasing global burden in solid organ and bone marrow transplant recipients, cancer patients, those with HIV, and those being treated with immunomodulators. The most common causes of IFD are *Candida* spp., followed by *Aspergillus* spp.; other pathogens such as *Cryptococcus* spp., the Mucorales, and *Pneumocystis* accounting for varying frequency of IFDs depending on geographic region and patient population (Brown et al., 2012). Despite advances in antifungal therapy, mortality rates from IFD are substantial but vary with infection. Increased prevalence, in particular of *Candida glabrata* infections including those due to azole-resistant, or echinocandin-azole co-resistant isolates, as well as multi-azole resistant *Candida tropicalis* isolates has been noted (Pfaller et al., 2012; Chapman et al., 2017). Of added concern is the global emergence of multi-drug resistant fungal species, including *Candida auris* (Desoubeaux et al., 2018), pan-azole resistant *Aspergillus fumigatus* driven by agricultural triazole use (Snelders et al., 2009; van der Linden et al., 2013; Navalkele et al., 2017), as well as rare molds which are often resistant to most if not all antifungal drugs (Malani and Kauffman, 2007; Dellière et al., 2019).

Early diagnosis of IFD including accurate identification of the causative fungus and where possible, of antifungal resistance, is critical for appropriate patient management and improving outcomes. While culture and microscopy remain the gold standard for IFD diagnosis, sensitivity and specificity of such methods are limited; cultures are slow (up to 4 weeks) and dependent on the specimen containing viable fungal elements. Fungi with environmentally and clinically acquired antifungal resistance are emerging, and cryptic species with intrinsic resistances may be missed. Therefore, there is a need for more sensitive and targeted diagnostic systems for IFD, to not only directly detect fungal species in clinical specimens, but also for more rapid detection of drug resistance. This article will focus on modern molecular diagnostic approaches to directly detect fungi in clinical specimens, and in the characterization of cultured fungi including their drug resistance profiles, with discussion on newer approaches including DNA barcoding and next generation sequencing (NGS). The review presents these approaches in the relevant clinical context, offers a broad view of where they may be positioned in a diagnostic laboratory, and concludes with the challenges that may be faced with wider implementation of molecular tests in an era where their use is envisaged to increase. As such, the consensus definitions for IFD from the European Organization for Research and Treatment of Cancer/Mycoses Special Interest Group (EORTC/MSG) have recently been updated to include some of the tests described here (Donnelly et al., 2019).

## Direct Detection of Fungi in Clinical Specimens

Molecular assays for the direct detection of fungal DNA in clinical specimens comprise either broad range (or panfungal) assays to capture “all fungi” or those tailored to detect specific genera or species. While increasingly used, their position in routine diagnostics will vary according to clinical context. Further, commercialization of assays has led to standardized methodologies, facilitating large scale “real world” clinical validation for routine clinical use (Zhang, 2013). The utility and challenges associated with both broad range and targeted genus-specific approaches are discussed in detail below.

### Panfungal PCR Assays

**FIGURE 1:**
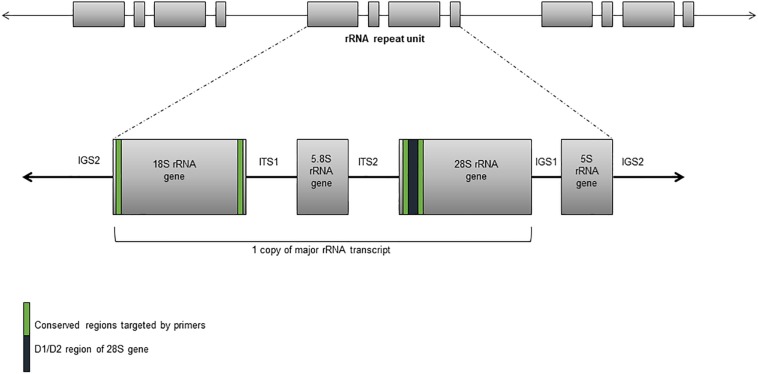
Ribosomal RNA gene cluster comprising the 18*S*, 5.8*S*, and 28*S* rRNA subunit genes, and separated by the internal transcribed sequences and the intergenic spacers.

These assays detect “all” fungal DNA present in a clinical specimen through the use of universal fungal primers. The preferred target(s) are one or more regions of the rRNA gene cluster – the internal transcribed spacers 1 and 2 (ITS1 and ITS2) and the D1/D2 regions of the 28*S* rRNA gene (Figure 1; White et al., 1990). Amplification is most often followed by DNA sequencing but high-resolution melt curve analysis in real-time PCR assays is increasingly used (Bezdicek et al., 2016; Valero et al., 2016). Together, these assays have successfully detected and identified fungi from diverse specimen types including fresh tissue, formalin fixed paraffin embedded (FFPE) tissue, cerebrospinal fluid (CSF), vitreous fluid, blood, and bronchoalveolar lavage fluid (BALF) (Lau et al., 2007; Landlinger et al., 2010; Bezdicek et al., 2016; Rahn et al., 2016; Rickerts, 2016; Valero et al., 2016; Gomez et al., 2017; Zeller et al., 2017; Sabino et al., 2019) with good accuracy and specificity though with varying sensitivity between specimen types. One study reported the best results when performed on sterile fluid specimens (including blood, CSF, and aspirates) with a sensitivity, specificity, negative predictive value (NPV), and positive predictive value (PPV) of 100, 96, 100, and 86%, respectively, but these values decreased to 90, 75, 86, and 82% from BALF (Zeller et al., 2017). An added advantage of such assays is that unexpected and novel pathogens may be identified. A prospective study of blood specimens from hematological patients at high risk for IFD reported 44.4% (8/18) of positive samples were identified as less common non-*Aspergillus* and non-*Candida* fungi including *Fusarium* spp., *Scedosporium apiospermum*, and various mucormycetes (Sugawara et al., 2013). In another study, panfungal PCR was able to provide an unexpected diagnosis of cerebral aspergillosis in a patient with osteosarcoma (Komatsu et al., 2004).

Gomez et al. (2017) investigated the diagnostic accuracy and clinical impact of an in-house panfungal sequence-based PCR assay in patients with a proven or suspected IFD. In patients with proven IFD (*n* = 60), the sensitivity and specificity were 96.6 and 98.2%, respectively, with similar assay sensitivities between different specimen types: 100% from both fresh tissue (*n* = 25) and sterile body fluids (*n* = 15), and 90% (18/20) from FFPE tissue. In patients with suspected IFD (*n* = 116), the diagnostic yield of the assay was 62.9% for all patients and 71.3% in patients with proven IFD. The sensitivity and diagnostic yield from this study are higher than reported by others (Lau et al., 2007; Rickerts et al., 2011; Trubiano et al., 2016) since testing was restricted to those specimens with visible fungal elements on histopathology, thereby increasing the pre-test probability of a positive result. Of note, the diagnostic yield was further improved in tissue specimens collected by open resection compared with core-needle biopsy and fine-needle aspiration highlighting the importance of evaluating sample volume adequacy to exclude false negative results. In addition, the inclusion of a human gene target such as β-actin as a DNA extraction control is essential (Gomez et al., 2017). The recently revised EORTC/MSG criteria for diagnosis of IFD from tissue specimens recommends the amplification of fungal DNA by PCR combined by DNA sequencing *only* when fungal elements are seen by histopathology (Donnelly et al., 2019).

Conversely, the interpretation of panfungal PCR results performed on non-sterile samples, particularly BALF, is more difficult (Halliday et al., 2015; Bezdicek et al., 2016; Rahn et al., 2016; Trubiano et al., 2016; Wehrle-Wieland et al., 2018). A positive result returned on BALF may indicate lung infection but also airway colonization or environmental contamination especially if non-pathogenic fungal species are detected. Indeed, in two studies, the most abundant species detected in BALF were *Candida* spp. (25–40%), followed by *Saccharomyces* spp. and *Rhodotorula* spp. (4–5% each) (Rahn et al., 2016; Trubiano et al., 2016). Sequencing the PCR product may also result in a mixed “signal” due to multiple fungal species being present (Bezdicek et al., 2016; Zeller et al., 2017); this can be partially overcome by post PCR high-resolution melt curve analysis rather than using sequencing (Bezdicek et al., 2016). The sensitivity of panfungal PCR on BALF is lower in patients receiving mold-active treatment (Trubiano et al., 2016; Wehrle-Wieland et al., 2018). While panfungal PCR on BALF has the potential to identify more fungal species than culture, a study by Bousbia et al. (2012) reported more episodes of pneumonia were diagnosed by culture (42%) than by molecular methods (17%).

From collective experience, it is clear that panfungal PCR has good utility where fungal elements are seen in fresh tissue and FFPE tissue by histopathological examination (i.e., proven IFD) (Donnelly et al., 2019) or where fungal hyphae/yeast forms are seen in sterile body fluids. Under these circumstances, panfungal PCR combined with DNA sequencing will strongly value add to the diagnosis by identifying the causative pathogen(s). The identity of the fungus should be consistent with the histopathologic or microscopic findings.

Where an IFD is in the differential diagnosis with a highly suggestive clinical picture, but where fungal forms are not visualized in the clinical specimen, e.g., to screen blood of patients at high risk for IFD, expert opinion suggests that panfungal PCR has good potential to assist with diagnosis by augmenting culture methods, although a negative result does not exclude diagnosis. This may be especially relevant in patients already receiving antifungal therapy (Trubiano et al., 2016; Ala-Houhala et al., 2018).

Until recently, a potential drawback of panfungal PCR methods was the lack of standardization of assays for comparison of results in the clinical trial or study context. The Fungal PCR Initiative (FPCRI) working group of the International Society for Human and Animal Mycoses (ISHAM) is currently designing and optimizing protocols to evaluate PCR methods to detect fungi from tissue specimens^1^. That acknowledged the use of such assays for routine patient care is often helpful in diagnosis and should be considered in the diagnostic algorithm of any patient with suspected IFD to reduce the possibility of misdiagnosis or missed diagnosis (Ullmann et al., 2018).

### *Aspergillus* PCR

For many years, the test performance of PCR-based assays for the diagnosis of invasive aspergillosis (IA) has varied substantively due to differences in methodology, sample type and volume, DNA extraction protocols, gene targets, differences in criteria to define “PCR positivity,” and reference standards to rule in, or rule out, infection (White et al., 2010a). Many of these studies focused on the use of *Aspergillus* PCR for the early or pre-emptive diagnosis of IA, i.e., in the screening of high risk hematology patients for IA. To this end, there have been a number of meta-analyses addressing these issues in *Aspergillus* PCR performance (Mengoli et al., 2009; Cruciani et al., 2015). A Cochrane library meta-analysis of PCR-based studies on blood specimens for early detection and diagnosis of IA (i.e., screening for infection) reported a mean sensitivity and specificity of 80.5% (95% CI 72.9–86.3%) and 78.5% (67.8–86.4%), respectively, for a single positive PCR result, and 57.9% (36.5–76.8%) and 96.2% (89.6–98.6%) for two positive PCR results (Cruciani et al., 2015). Assuming a mean prevalence of IA of 13% in a particular patient population, the PPV of *Aspergillus* PCR increased from 36 to 70% when two positive results were used to define a “PCR positive” episode, while the NPV remained at 96 to 94%. The high NPV of *Aspergillus* PCR allows IA to be ruled out in the presence of a negative test result with little need for empiric antifungal therapy (Cruciani et al., 2015; Pasqualotto and Falci, 2016). These results were confirmed in a large prospective study to determine the efficacy of PCR screening for early diagnosis of IA in 213 high risk patients undergoing hematopoietic stem cell transplantation or chemotherapy for acute leukemia (Springer et al., 2016a). In antifungal drug-naïve patients, the sensitivity, specificity, PPV, and NPV of PCR were 71.4, 92.3, 62.5, and 98.3%, respectively. However, the PPV decreased to only 5.4% (the NPV rose to 100%) in patients receiving *Aspergillus*-active prophylaxis, suggesting the role of screening by PCR is best applied to patients not receiving primary anti-mold prophylaxis (Springer et al., 2016a).

To harmonize test performance between studies, the European *Aspergillus* PCR Initiative (EAPCRI) Working Group of ISHAM has standardized *Aspergillus* PCR methodology for analytical performance and clinical validity^2^. Laboratories performing *Aspergillu*s PCR should employ these recommendations when performing PCR in whole blood (WB), serum, and plasma (White et al., 2010a, b, 2011, 2015; Loeffler et al., 2015); validation of similar standardized processes using BALF is underway (Barnes et al., 2018). Although data are lacking for validation of CSF, urine, and tissue samples, it is likely that the same principles and critical steps will apply (i.e., sample volume ≥ 0.5 mL and elution volume < 100 μL) (White et al., 2010a; Barnes et al., 2018).

Debate is ongoing as to whether WB, serum, or plasma specimens should be used for screening or to diagnose IA by PCR (Buchheidt et al., 2017). Processing of serum or plasma, compared with WB, is technically less demanding as it facilitates automated processing, requires less standardization, and has reduced risk of contamination (White et al., 2011, 2015; Loeffler et al., 2015). A study that applied EAPCRI-standardized methodologies to evaluate all three blood fractions (423 WB, 583 plasma, and 419 serum) found that for all samples, PCR positivity was associated with cases of IA (plasma, *P* = 0.0019; serum, *P* = 0.0049; and WB, *P* = 0.0089). Plasma PCR generated the highest sensitivity (91%) compared with serum (80%) and WB (55%); however, the specificity for WB (96%) was significantly higher than those of serum (69%, *P* = 0.0238) or plasma (53%, *P* = 0.0002) (Springer et al., 2016b). Further combining PCR testing of different blood fractions allowed IA to be both excluded and diagnosed. Testing of plasma provides similar diagnostic utility to WB while allowing utilization of commercial automated DNA extraction processes more suited to routine laboratories (Loeffler et al., 2015; Springer et al., 2016b). It is noteworthy that *Aspergillus* PCR has been included as a mycologic criterion for probable IA in the revised EORTC/MSG definitions (Donnelly et al., 2019). The EAPCRI have recommended that *Aspergillus* genus-specific PCR assays targeting the rRNA genes are preferred for reliably detecting *A. fumigatus* at low DNA concentrations but thus far do not recommend a specific quantitative PCR (Morton et al., 2017).

For pragmatic reasons, it is reasonable for clinical mycology laboratories to test one blood fraction rather than two with the above caveat in mind. On balance, because the use of plasma lends itself to automated DNA extraction, this work-flow advantage is suited to busy clinical laboratories without compromising test performance. Sample volume (≥0.5 mL of plasma) and elution volume (<100 μL) are both critical.

Other than screening for early IA, the use of PCR to detect *Aspergillus* DNA in other clinical specimens (e.g., BALF, CSF, and tissue) for diagnosis in patients suspected to have IA has also been useful. However, as for panfungal PCR, BALF-PCR cannot distinguish colonization from IA resulting in relatively low PPV of around 72% (even lower in non-hematology patients) (Arvanitis and Mylonakis, 2015). Consequently, the use of PCR to diagnose IA and CNS aspergillosis or meningitis from BALF and CSF, respectively, is only moderately recommended by the European Society for Clinical Microbiology and Infectious Diseases (ECSMID), European Confederation of Medical Mycology (ECMM), and European Respiratory Society (ERC) joint guidelines (Ullmann et al., 2018); however, a negative PCR result is useful to exclude IA (Heng et al., 2014). The added inherent variability of BALF sampling procedures means that quantification of fungal burden by PCR cannot be interpreted meaningfully (Buchheidt et al., 2017). One study (Imbert et al., 2018) reported that determining fungal load by PCR in BALF allowed discrimination between aspergillosis and non-aspergillosis pathologies (i.e., contamination), but not between invasive and non-invasive forms. Several meta-analyses using PCR on BALF IA diagnosis reported overall sensitivities and specificities > 90% (Sun et al., 2011; Avni et al., 2012) and if EORTC/MSG criteria were strictly applied, the sensitivity and specificity were 77 and 94%, respectively (Avni et al., 2012). As for blood, antifungal treatment before bronchoscopy significantly reduced sensitivity (Avni et al., 2012).

*Aspergillus* PCR assays therefore have established utility in screening for early infection in high risk patients as well as being used in the diagnosis of established infection in real time. Greater clinical application may in the near future be realized by the growing number of commercial PCR assays to detect *Aspergillus* DNA in clinical specimens, providing standardized methodology, and quality control of the reagents (see Table 1). While the sensitivity and specificity of commercial assays are encouraging, data evaluating their clinical utility and head-to-head comparisons are relatively limited (Danylo et al., 2014; Chong et al., 2015; White et al., 2017b; Barnes et al., 2018; White, 2019). A recent review of commercial assays for the detection of *Aspergillus* spp. reported significantly lower sensitivities and specificities in serum specimens than respiratory specimens. Only the MycAssay *Aspergillus*® (Microgen Bioproducts Ltd., Camberley, United Kingdom) and the AsperGenius®(PathoNostics, Maastricht, Netherlands) assays were recommended for routine testing of respiratory samples (Rath and Steinmann, 2018), although the MycAssay *Aspergillus* is no longer commercially available. No recommendations were made for routine testing of serum specimens using commercial assays. Some assays simultaneously detect both *Aspergillus* DNA and the most prevalent *CYP51A* gene mutations responsible for azole resistance in *A. fumigatus*, differentiating wild type from resistant strains (see section “Molecular Detection of Antifungal Drug Resistance”).

**TABLE 1:** List of commercially available PCR-based assays for detection of *Aspergillus* spp. and *CYP51A* resistance mutations in *A. fumigatus*.

**Product**	**Manufacturer**	**Method**	**PCR target,^∗^ species, and resistance mutations detected**
Affigene *Aspergillus* tracer	Cepheid, Rolling Meadows, IL, United States	Real-time PCR	Target unknown *Aspergillus* spp.
*A. fumigatus* Bio-Evolution	Bio-Evolution, Bry-sur-Marne, France	Real-time PCR	ITS1 region *A. fumigatus*
artus® Aspergillus diff. RG PCR	Qiagen, Düsseldorf, Germany	Multiplex real-time PCR	Target unknown *A. fumigatus*, *A. terreus*, *A. flavus*
AsperGenius® Species and AsperGenius® Resistance	PathoNostics B.V., Maastricht, Netherlands	Multiplex real-time PCR	28*S* rDNA *A. fumigatus* complex, *A. terreus*, *Aspergillus* spp. TR_34_/L98H and TR_46_/Y121F/T289A mutations
*Aspergillus* spp. ELITe MGB® Kit	ELITechGroup S.p.A, Turin, Italy	Quantitative real-time PCR	18*S* rDNA *Aspergillus* spp. (*A. niger*, *A. nidulans*, *A. terreus*, *A. flavus*, *A. versicolor*, *A. glaucus*)
*Asp*ID	OlmDiagnostics, Newcastle, United Kingdom	Multiplex real-time PCR	Target unknown *Aspergillus* spp., *A. terreus*
FungiPlex® *Aspergillus* and Fungiplex® *Aspergillus* Azole_R	Bruker Daltonik GmbH, Bremen, Germany	Multiplex real-time PCR	Target unknown *Aspergillus* spp. (*A. fumigatus*, *A. flavus*, *A. niger*), *A. terreus* TR_34_ and TR_46_ mutations
LightCycler Septifast	Roche Diagnostics, Mannheim, Germany	Multiplex real-time PCR	ITS region *A. fumigatus* (and *Candida* spp.)
Magicplex Sepsis Real-Time Test	Seegne, Seoul, South Korea	Multiplex real-time PCR assay	Target unknown *A. fumigatus* (and *Candida* spp.)
MycoReal Aspergillus	Ingenetix GmbH, Vienna, Austria	Real-time PCR with melt curve analysis	ITS2 region *A. fumigatus*, *A. flavus*, *A. nidulans*, *A. niger*, *A. terreus*
MycoGENIE® *Aspergillus* Species and MycoGENIE® *Aspergillus fumigatus* and resistance TR_34_/L98H	Ademtech, Pessac, France	Duplex real-time PCR assay	28*S* rDNA *Aspergillus* spp., *A. fumigatus* TR_34_/L98H mutations

*^∗^PCR target where specified by the manufacturer. Reviewed by Rath and Steinmann (2018).*

### PCR for Invasive Candidiasis

Definitive treatment of invasive candidiasis (IC), encompassing candidemia and deep-seated candidiasis, is often delayed by the insensitivity of culture with high mortality (35–75%) (Fortún et al., 2012). Blood cultures are sensitive at detecting viable *Candida* cells, with a limit of detection of ≤ 1 colony forming unit (CFU)/mL, but their overall sensitivity across the spectrum of IC is only ∼50% and they have a lag time for identification of up to 5 days (Pfeiffer et al., 2011; Clancy and Nguyen, 2013). Time to initiation of appropriate antifungal therapy and to source control are critical determinants of survival (Clancy and Nguyen, 2018a). The high sensitivity of PCR-based assays, detecting < 5 CFU/mL, makes these appealing for early diagnosis of IC, particularly those cases of IC that are missed by culture (Pfeiffer et al., 2011; Clancy and Nguyen, 2013), yet the position of *Candida* PCR assays in the diagnostic algorithm of IC is not as clear as that of *Aspergillu*s PCR (discussed above), and its use will differ in different clinical contexts.

A major impediment to clinical application has been lack of standardization, variable analytical sensitivity, and the need for nucleic acid extraction and purification from clinical samples (Pfaller and Castanheira, 2016). Numerous DNA extraction methods and use of various gene targets [predominantly rDNA sequences and cytochrome P450 lanosterol 14 alpha-demethylase (*L1A1*) gene], PCR formats (single and multiplex, nested, and real-time PCR assays), both in-house and commercial, have been evaluated for the rapid diagnosis of IC (Arvanitis et al., 2014). These differences in methodologies and case definitions have limited comparison between studies. Most *Candida* PCR data have been sourced using WB or blood fractions with reported high sensitivities (80–100%), specificities (90–100%), and NPVs (88–100%) for those species targeted. This suggests that PCR-based assays may be more useful in excluding, rather than establishing, the diagnosis of IC (Arvanitis et al., 2014; Fortún et al., 2014). However, the clinical utility of these assays is uncertain due to their limited validation in real-life prospective settings.

A meta-analysis of 54 *Candida* PCR studies (>4600 patients) reported pooled sensitivities and specificities for detecting candidemia of 95 and 92%, respectively, with results available up to four weeks earlier than standard culture or clinical signs of IC (Avni et al., 2011). In patients with probable IC, the positivity rate of PCR was 85% compared with 38% for blood cultures. The specificity was > 90% for the same patients but decreased among *Candida*-colonized controls. Improved test performance was associated with the use of WB (rather than serum) and using a PCR targeting a *Candida*-specific part of the rDNA or cytochrome P450 genes with an *in vitro* detection limit of ≤ 10 CFU/mL. For blood specimens, studies using real-time PCR and species-specific probes have shown the best results. For IC caused by both *Candida albicans* and non-*C*. *albicans* species, McMullan et al. (2008) reported sensitivity, specificity, PPV, and NPVs of 87, 100, 100, and 99.6%, respectively, for the six species detected (*C. albicans*, *C. tropicalis*, *Candida parapsilosis*, *Candida dubliniensis*, *Candida glabrata*, and *Candida krusei*).

PCR performance data for forms of IC other than candidemia are relatively limited – for intra-abdominal candidiasis, PCR sensitivities of 86–91% have been reported for the species targeted, but specificity varies widely (33–97%) (Nguyen et al., 2012; Fortún et al., 2014; León et al., 2016; Clancy and Nguyen, 2018b). Nguyen et al. (2012) found a *Candida* real-time PCR assay to be more sensitive than the Fungitell® 1,3-β-D glucan (BDG) assay (Associates of Cape Cod, United States) for diagnosis of all IC (80 versus 56%) as well as deep seated (blood culture negative) candidiasis (89 versus 53%). Both PCR and BDG were more sensitive than blood cultures for diagnosis of deep-seated candidiasis (sensitivities of 88, 62, and 17%, respectively). However, if blood culture was combined with either PCR or BDG, the sensitivities increased to 98 and 79%, respectively. Data on the application of *Candida* PCR assays are almost exclusively limited to blood specimens; however, it was recently reported to be useful in the diagnosis of *Candida* meningitis, which has a poor yield from CSF culture (Herrera et al., 2019).

A number of commercial PCR-based diagnostic methods are now available for IC (Table 2). Generally, these assays target the five most common pathogenic *Candida* species (*C. albicans*, *C. glabrata*, *C. krusei*, *C. parapsilosis*, and *C. tropicalis*) which account for > 95% of IC. Many of these assays remain investigational as they have not been validated for diagnosing IC in multi-center studies, nor is there evidence that any one commercial test is superior. The choice of adopting an in house rather than commercial assay is dependent upon workflow and capacity in individual testing laboratories and cost (Clancy and Nguyen, 2018b), and results should be considered with caution. To date, the T2Candida panel and the T2Dx instrument (T2 Biosystems, United States) is the only platform approved by the US Food and Drug Administration (FDA) to detect *Candida* spp. in WB without the need for prior blood culture or nucleic acid extraction steps. The platform combines nuclear magnetic resonance and PCR technology for rapid (< 3 h), accurate, sensitive (1–3 CFU/mL), and specific detection of *C. albicans*/*C. tropicalis*, *C. parapsilosis*, and *C. glabrata*/*C. krusei* (Neely et al., 2013; Mylonakis et al., 2015). The subsequent T2 *C. auris* panel detects *C. auris* a limit of detection < 5 CFU/mL from WB and skin swabs in under 5 h (Sexton et al., 2018).

**TABLE 2:** List of commercially available PCR-based assays for detection of *Candida* spp.

**Product**	**Manufacturer**	**Method**	**PCR TARGET^∗^ and species detected**
AusDiagnostics Sepsis panel	AusDiagnostics Pty Ltd., Mascot, NSW, Australia	Multiplex tandem PCR	ITS1 or ITS2 regions *C. albicans*, *C. glabrata*, *C. krusei*, *C. parapsilosis*, *C. tropicalis*
*Cand*ID® and *Auris*ID®xs	OlmDiagnostics, Newcastle, United Kingdom	Multiplex real-time PCR	Target unknown *C. albicans*, *C. dubliniensis*, *C. glabrata*, *C. krusei*, *C. parapsilosis*, and *C. tropicalis Candida auris*
FilmArray Blood Culture Identification (BCID) Panel^1^	BioFire Diagnostics, Salt Lake city, Utah, United States	Multiplex real-time PCR assay	Target unknown *C. albicans*, *C. glabrata*, *C. krusei*, *C. parapsilosis*, and *C. tropicalis*
FungiPlex® Candida and FungiPlex® Candida auris	Bruker Daltonik GmbH, Bremen, Germany	Multiplex real-time PCR assay	Target unknown *Candida* spp. (*C. albicans*, *C. parapsilosis*, *C. dubliniensis*, *C. tropicalis*), *C. glabrata*, and *C. krusei Candida auris*
Magicplex Sepsis Real-Time Test	Seegne, Seoul, South Korea	Multiplex real-time PCR assay	Target unknown *C. albicans*, *C. glabrata*, *C. krusei*, *C. parapsilosis*, and *C. tropicalis* (and *A. fumigatus*)
MycoReal Candida	Ingenetix, Vienna, Austria	Real-time PCR with melt curve analysis	ITS2 region *C. albicans*, *C. dubliniensis*, *C. glabrata*, *C. krusei*, *C. lusitaniae*, *C. parapsilosis*, and *C. tropicalis*
SeptiFast Real-Time PCR	Roche Diagnostics, Mannheim, Germany	Multiplex real-time PCR assay	Target unknown^∗^ *C. albicans*, *C. glabrata*, *C. krusei*, *C. parapsilosis*, and *C. tropicalis*
SepsiTest-UMD	Molzym Molecular Diagnostics, Bremen, Germany	PCR and Sanger sequencing	18*S* rDNA All fungal species
T2Candida	T2 Biosystems, Lexington, MA, United States	T2 magnetic resonance	ITS2 region *C. albicans*/*C. tropicalis*, *C. glabrata* complex/*C. krusei*, and *C. parapsilosis* complex
Sepsis Flow Chip	Master Diagnostica, Granada, Spain	Multiplex PCR with automated reverse dot blot hybridization	Target unknown^∗^ *C. albicans*, *Candida* spp.

*^∗^PCR target where specified by the manufacturer. ^1^Requires a positive blood culture.*

In the initial multi-center trial of T2 Biosystems (DIRECT), using spiked blood samples from hospitalized patients, the assay had a sensitivity and specificity of 91.1 and 99.4%, respectively, with a time to positivity of 4.4 ± 1 h (Mylonakis et al., 2015). In a follow-up multi-center trial (DIRECT2) of hospitalized patients with candidemia, the clinical sensitivity for the five species identified by T2Candida was 89% as 32/36 patients with positive companion blood cultures (cBCs) yielded positive T2Candida results. T2Candida was significantly more likely to be positive than cBCs (45 versus 24%, *P* < 0.0001) (Clancy et al., 2018). Another study found T2Candida was clinically more effective than blood culture for the management of suspected candidemia, but slightly less effective than empiric therapy (Walker et al., 2016). Prospective randomized controlled trials are required to fully evaluate the impact of the T2Candida on patient outcomes and hospital-associated costs. In a setting with a prevalence of candidemia of 10%, the T2Candida is expected to have a PPV and NPV of 84 and 99%, respectively; however, in low-prevalence settings (0.4–1%), the PPV drops to an estimated 15–31% and a positive result by itself would be unlikely to justify antifungal treatment in a patient without identifiable risks or candidemia (Clancy and Nguyen, 2018c; Zacharioudakis et al., 2018). The performance of T2Candida in cases of deep-seated candidiasis has not yet been evaluated.

From the above data, for established candidemia and IC, *Candida* PCR has a higher sensitivity than blood culture but remains best positioned for use in conjunction with blood cultures and/or BDG testing. *Candida* PCR with its high NPV value, however, lends itself as a potential tool to rule out IC in high risk patients.

### PCR Detection of Mucormycosis

Invasive fungal diseases caused by Mucorales fungi are increasing, especially among immunocompromised patients and in those with poorly controlled diabetes mellitus (Vallabhaneni et al., 2017; Millon et al., 2019). The growing incidence of mucormycosis can in part be attributed to changes in antifungal protocols, particularly for the prevention of IA in high risk groups. But in practice, the increasing use of molecular techniques for the direct detection of Mucorales DNA in fresh and FFPE tissue that are often culture negative has likely led to an increase in diagnosed cases (Millon et al., 2019). Diagnosis of mucormycosis remains challenging as clinical features are non-specific and diagnosis often relies on standard laboratory methods which are non-specific and insensitive. Additionally, there are no commercially available serological tests for Mucorales (Lackner et al., 2014).

Conventional and real-time PCR assays for the direct detection of minute amounts of Mucorales DNA from fresh or FFPE tissue, BALF, and serum have been reported with the majority targeting the ITS, 18*S*, or 28*S* rDNA (Hammond et al., 2011; Millon et al., 2013, 2015; Lackner et al., 2014; Springer et al., 2016a, b; Gholinejad-Ghadi et al., 2018). The key to effective molecular diagnosis, however, is efficient DNA extraction from the clinical specimen. To date, PCR detection from fresh or FFPE tissue has been the preferred method to identify the causative mucormycete with sensitivities in FFPE tissue of 56–91% (Springer et al., 2016b; Gholinejad-Ghadi et al., 2018). Negative results from FFPE samples may be due to presence of very small amounts of fungal DNA and/or cross-linking of proteins and fragmentation of DNA during formalin fixation (Gholinejad-Ghadi et al., 2018).

More recently, advances have been made in the detection of Mucorales DNA by real-time qPCR in serum and on average, can detect the pathogen 8 days (up to 24 days) earlier than histology and/or culture and 3 days earlier than radiographic features such as reverse halo signs on CT scans (Millon et al., 2013, 2015; Caillot et al., 2016). Each of these studies used three qPCR assays targeting 18*S* rDNA from *Mucor/Rhizopus*, *Lichtheimia*, and *Rhizomucor* species. Not unexpectedly the sensitivity of qPCR on serum decreased upon receipt of active antifungal therapy (Ambisome alone or in combination with posaconazole) (Caillot et al., 2016). Springer et al. (2016b) recently determined the clinical value of screening serum samples (*n* = 268) from 35 high risk patients using a Mucorales-specific real-time PCR assay; an additional step of sequencing is required to identify the genus. Mucorales DNA was detected in sera from all patients with probable/proven (*n* = 5) and in 29% of patients (5/17) with possible mucormycosis. A screening approach would have enabled earlier diagnosis (up to 21 days) and targeted treatment in 80% of probable/proven cases.

qPCR detection of Mucorales DNA in BALF is likewise an attractive approach to aid the diagnosis of pulmonary mucormycosis. In a study by Scherer et al. (2018), Mucorales PCR was positive on BALF of all 10 patients with mucormycosis (seven proven and three probable). For four of these patients, PCR on BALF was the earliest available biological test revealing mucormycosis, and three of these four patients had already been diagnosed with aspergillosis. Mucorales–*Aspergillus* mixed infection is also thought to be more frequent than previously described (Scherer et al., 2018). These results provide support for Mucorales PCR on BALF to be included in the diagnostic approach to pulmonary IFD, although it is still difficult to obtain BALF in at risk hematology or ICU patients. A prospective multi-center trial of molecular tools for the early diagnosis of mucormycosis is underway (ModiMucor [Projet Hospitalier de Recherche Clinique] national-ModiMucor-French Ministry of Health 2014-A00580-47^3^).

The overall low number of patients studied and lack of standardization and thorough clinical evaluation of these in-house PCR assays means they cannot be recommended as stand-alone diagnostic tests they are valuable add-on tools that complement histology and culture (Cornerly et al., 2014; Lackner et al., 2014). The ISHAM FPCRI Mucorales Lab working group has been established to improve standardization and provide recommendations for Mucorales PCR (Millon et al., 2019). The recent development of a commercial real-time PCR assay for the detection of the clinically relevant Mucorales species by PathoNostics (Maastricht, Netherlands) should also assist the standardization of Mucorales PCR. This assay enables direct detection of pan-*Mucorales* DNA, *Rhizopus* spp., *Mucor* spp., *Lichtheimia* spp., *Cunninghamella* spp., and *Rhizomucor* spp. in respiratory tract samples and fresh and FFPE tissue within 3.5 h (Gaajetaan et al., 2018). It is currently available for “research use only” purposes.

Of note, the recent discovery of the gene family of spore coating encoding proteins (*CotH*) offers a novel potential diagnostic target. *CotH* genes are multi-copy and are universally and uniquely present in Mucorales fungi, allowing them to penetrate host cells. Baldin et al. (2018) investigated whether a *CotH*-specific PCR assay could detect DNA from a variety of Murorales species and genera (*Lichtheimia corymbifera*, *Rhizopus delemar*, *Rhizopus arrhizus*, *Mucor circinelloides*, and *Cunninghamella bertholletiae*) in various biological fluids (plasma, urine, and BALF) in mice. *CotH* DNA was detected with 100% specificity from all fluids within 24 h of infection but was more consistently detected in urine (90% sensitivity) than in plasma or BALF. PCR was also successful from urine samples of four patients with proven mucormycosis, showing the potential of *CotH* genes as a biomarker for this and warranting validation on larger numbers of human samples (Baldin et al., 2018).

### *Pneumocystis jirovecii* PCR

PCR-based assays for the diagnosis of *Pneumocystis jirovecii* pneumonia (PCP) undoubtedly are more sensitive than histological and microscopic identification of asci and trophic forms in tissue, BALF, and induced sputum (IS) by conventional staining or by immunofluorescence microscopy (White et al., 2017a). Numerous comparative studies have been published (beyond the scope of this review); however, a recent comparison shows an in-house qPCR method had sensitivity of 82.9% compared to 60.0% for the monoclonal immunofluorescence assay by the MonoFluo^TM^ *P. jirovecii* IFA test kit (Bio-Rad, Marnes-la-Coquette, France) with similar specificity (>99%) (Desoubeaux et al., 2017). For pragmatic purposes, for more rapid TATs and because of steady loss of microscopy skills in the mycology laboratory, an increasing number of laboratories are turning to *Pneumocystis* PCR assays. However, as PCR may detect colonization, asymptomatic infection, subclinical infection, as well as active infection, this raises the issue of interpretation of discordant results, e.g., between a PCR positive and immunofluorescence negative sample, or indeed of any “positive” PCR result.

Three meta-analyses, each of > 400 cases of PCP, demonstrated the excellent performance of PCR for both diagnosis and exclusion of PCP with positive and negative likelihood ratios of ≥ 10 and ≤ 0.3, respectively and sensitivities, specificities, PPVs, and NPVs ranging from 97–99, 90–94, 66–85, and > 99%, respectively (Lu et al., 2011; Fan et al., 2013; Summah et al., 2013). Hence to exclude *Pneumocystis* colonization, positive PCR results should be interpreted in conjunction with clinical, radiological, and laboratory findings. qPCR assays gave better sensitivities and specificities than non-qPCR assays (Lu et al., 2011; Fan et al., 2013; Maillet et al., 2014), and are recommended for the routine diagnosis of PCP due to their potential for distinguishing colonization from infection, although guidelines for this are lacking (Alanio et al., 2016). The recently revised EORTC/MSG definitions include detection of *P. jirovecii* DNA in a respiratory tract specimen by qPCR as mycological evidence of pneumocystosis but do not recommend a threshold for positivity (Donnelly et al., 2019).

Bronchoalveolar lavage fluid is the preferred specimen for PCP diagnosis (Alanio et al., 2016). However, since it is not always possible to obtain BALF, upper respiratory tract specimens including IS, oropharyngeal washings (OW), nasopharyngeal aspirates (NA), and nasal swabs (NS) have been evaluated. Strict comparisons between different respiratory specimens are scarce but generally the yield from BALF is greater than IS, which in turn is greater than that from OW (Alanio et al., 2016). Lu et al. (2011) showed OW had significantly lower sensitivity (76%) although higher specificity (93%) compared with BALF (100 and 88%, respectively) but the positive likelihood ratio of 10.4 for OW, compared with 8.0 in BALF, indicated that detection of *Pneumocystis* DNA in upper respiratory tract specimens can indicate PCP.

Alanio et al. (2011) attempted to establish qPCR cut-off values to differentiate true disease from colonization. They reported no significant difference in fungal DNA load between IS and BALF samples and that similar cut-off values were applicable to both. However, there were significantly higher fungal burdens in BALF and IS from HIV-infected patients compared with those from non-HIV-infected immunocompromised patients. Fauchier et al. (2016) demonstrated different qPCR cycle thresholds (CT) to exclude PCP colonization from infection were applicable to HIV-infected and non-HIV-infected patients. Applying different CT values to the two patient groups was associated with 100% specificity for the diagnosis of PCP in the HIV-positive patients but only 80% in non-HIV patients, i.e., PCP colonization was a possibility for 20% of this group, limiting the clinical value of the assay.

When attempting to use a CT value to differentiate infection from colonization, the quality of the specimen must also be considered. Theoretically determining the quantity of human DNA can act as a surrogate for assessing sample quality, although for reference it is essential to know the typical burden of human DNA in respiratory samples and that sampling of respiratory specimens is standardized, which is not the case for BALF (White et al., 2017a). If technical sources of false negatives have been eliminated, a negative qPCR can be used to rule out PCP in BALF, but cannot exclude disease when used on IS, sputa, or other upper respiratory tract specimens (Alanio et al., 2016).

Several commercial qPCR assays for PCP detection are available but there are few comparative studies. A performance evaluation of three kits (AmpliSens *P. jirovecii*-FRT, MycAssay *Pneumocystis* from Myconostica, and Bio-Evolution *P. jirovecii* PCR) together with an in-house assay targeting the major surface glycoprotein gene found excellent concordance between the in-house assay, Ampli-Sens, and MycAssay (kappa, > 0.8), with all three assays confirming the diagnosis in 100% of proven (*n* = 12) and probable (*n* = 25) PCP groups, compared with 100 and 92%, respectively, of cases confirmed by the Bio-Evolution assay (Sasso et al., 2016). The percentage of positive results was more variable for the “possible PCP” category, ranging from 54.5% (Bio-Evolution) to 86.4% (AmpliSens PCR), but all four assays were effective for PCP diagnosis. More recently, the performance of the RealStar *P. jirovecii* PCR Kit 1.0 EC (Altona Diagnostics, Hamburg, Germany) was compared with the AmpliSens PCR assay (Huh et al., 2019). The positive and negative percent agreements were 100 and 96.6%, respectively, and kappa was 0.92. In contradiction to Alanio et al. (2011), this study demonstrated low detection rates from sputum and endotracheal aspirates compared with BALF.

Of interest, the PneumoGenius® real-time PCR assay (PathoNostics) combines *P. jirovecii* amplification with detection of point mutations in the dihydropteroate synthase (*DHPS*) gene. A study evaluating the performance of this assay for distinguishing probable from unlikely PCP reported a sensitivity and specificity of 70 and 82%, respectively (Montesinos et al., 2017), further observing good correlation between the PneumoGenius®, an in-house assay qPCR, and the Bio-Evolution qPCR. The DHPS genotype of 25% (31/120) of PCR-positive samples could not be determined, likely due to the low fungal burden observed. Nevertheless, the assay showed a 4.5% resistance rate from 89 samples. The clinical significance of mutations in the DHPS gene remains a matter of debate. While studies have demonstrated an association between the use of sulfa drugs for PCP prophylaxis and DHPS mutations, whether these mutations confer resistance to sulfamethoxazole, for PCP treatment is unclear as published studies provide conflicting results (summarized in Huang et al., 2004; Alvarez-Martínez et al., 2010). Clinical resistance to sulfonamides in *P. jirovecii* is very uncommon.

The *Pneumocystis* working group of the FPCRI was established to evaluate the performance of the different PCP PCR assays used in reference laboratories in order to establish a consensus method for qPCR and assist laboratory standardization of quantification. An intra-laboratory analysis compared five in-house and five commercial qPCR assays. Assays targeting whole nucleic acid (RNA plus DNA) using reverse transcriptase (RT)-qPCR were superior to qPCRs (*p* ≤ 0.001) testing DNA only, and assays targeting the multi-copy mitochondrial small subunit (mtSSU) were more sensitive than the mitochondrial large subunit (mtLSU), the major surface glycoprotein, and the single copy beta-tubulin gene (Gits-Muselli et al., 2019).

### Syndromic Testing

The use of syndromic testing PCR panels for diagnosis of various infections is increasingly popular. For IFDs, there are a number of FDA – or comparable regulatory agency – approved panels. As part of the target menu for CNS infections, the BioFire FilmArray Meningitis/Encephalitis panel (bioMérieux, Marcy l’Etoile, France) detects one fungal target, *Cryptococcus neoformans/Cryptococcus gattii*, in addition to bacterial and viral targets in CSF in approximately 1 h. While rapid, this test does not differentiate between *C. neoformans* and *C. gattii*, and recent studies have noted poor detection of cryptococcal infection when compared with cryptococcal antigen tests (Liesman et al., 2018; Lee et al., 2019). Test costs are also high (about AUD 250/test). A multiplex tandem-PCR (MT-PCR) based panel, also for use on CSF (AusDiagnostics, Mascot, Australia) includes a target that detects the four main serotypes of *C. neoformans* species complex. Data on the performance of this test in the clinical setting are few.

For patients with suspected fungal pneumonia, the AusDiagnostics Pneumonia and Atypical Pneumonia MT-PCR panels (AusDiagnostics) include a range of bacterial and viral targets, as well as one for *A. fumigatus* (RUO), *P. jirovecii*, and *C. neoformans/gattii* (Table 3). These panels utilize a range of respiratory specimens including sputum, bronchial washings, and BALF. There are no published evaluations of this test.

**TABLE 3:** List of commercially available PCR-based assays for detection of Mucorales and *Pneumocystis jirovecii*.

**Product**	**Manufacturer**	**Method**	**PCR target,^∗^ species, and resistance mutations detected**
MucorGenius®	PathoNostics B.V., Maastricht, Netherlands	Multiplex real-time PCR	Target unknown Pan-Mucorales, *Rhizopus* spp., *Mucor* spp., *Lichtheimia* spp., *Cunninghamella* spp., and *Rhizomucor* spp.
AmpliSens *Pneumocystis jirovecii* (*carinii*)-FRT	AmpliSens, Bratislava, Slovak Republic	Real-time PCR	Target unknown *Pneumocystis jirovecii*
AusDiagnostics Pneumonia and Atypical Pneumonia panels	AusDiagnostics Pty Ltd., Mascot, NSW, Australia	Multiplex tandem PCR	Targets unknown *Pneumocystis jirovecii*, *Cryptococcus neoformans* species complex, and *Aspergillus fumigatus* complex
PneumoGenius	PathoNostics B.V., Maastricht, Netherlands	Multiplex real-time PCR	Mitochondrial ribosomal large subunit (rLSU) and two dihydropteroate synthase (DHPS) *fas* gene mutations *Pneumocystis jirovecii* and point mutations: 165 (Thr55Ala) and 171 (Pro57Ser)
*Pneumocysist jiorovecii* Bio-Evolution	Bio-Evolution, Bry-sur-Marne, France	Real-time PCR	Target unknown *Pneumocystis jirovecii*
PneumoID®	OlmDiagnostics, Newcastle, United Kingdom	Multiplex real-time PCR	Target unknown *Pneumocystis jirovecii*
Real Star Pneumocystis jirovecii PCR kit 1.0	Altona Diagnostics, Hamburg, Germany	Real-time PCR assay	Target unknown *Pneumocystis jirovecii*

*^∗^PCR target where specified by the manufacturer.*

Lastly, a number of “sepsis” syndromic panels have been developed. Again, one from AusDiagnostics which works with nucleic acid extracts from positive blood cultures includes five *Candida* species among its 24 targets (Table 2). The ePlex assays (GenMark Diagnostics, Carlsbad, CA, United States) are fully automated tests based on electrowetting using eSansor technology for the detection of analyte targets directly from positive blood cultures after multiplex nucleic acid extraction, amplification, and digestions. Cartridges for the identification of Gram negative bacteria (BCID-GN), Gram positive bacteria (BCID-GP), and fungal pathogens (BCID-FP) directly from positive blood culture bottles may be applied as required, depending on Gram stain results; the BCID-FP panel targets 16 fungal species/genera, including *C. albicans*, *C. auris*, *C. dubliniensis*, *Candida famata*, *C. glabrata*, *Candida guilliermondii*, *Candida kefyr*, *C. krusei*, *Candida lusitaniae*, *C. parapsilosis*, *C. tropicalis*, *Cryptococcus gattii*, *Cryptococcus neoformans*, *Fusarium* spp., and *Rhodotorula* spp. (Maubon et al., 2018). A small validation study found the BCID-FP panel had 100% sensitivity and 100% specificity for six *Candida* species grown from blood cultures, which the panel was designed to detect. However, one case of *Candida inconspicua* (not targeted by the BCID-FP panel) was missed. The combination of blood culture and ePlex reduced the turn-around time from 72–96 to 10 h (Huang et al., 2019). The Accelerate Pheno system (Accelerate Diagnostics) is a fully automated system utilizing gel electrofiltration and fluorescence *in situ* hybridization (FISH) for the identification of two *Candida* species as well as bacteria, directly from positive blood cultures. This system had sensitivities of 100% for both *Candida* sp., and specificities of 100 and 97.8% for *C. albicans* and *C. glabrata*, respectively. The time to identification is approximately 90 min (Charnot-Katsikas et al., 2017).

All syndromic test panels require larger scale evaluation to determine their position in the routine laboratory. Given that these syndromic panels are designed to detect a limited range of specific pathogens, their usefulness may be limited to specific populations, e.g., emergency departments, or to specific setting, e.g., suspected meningitis, where a rapid answer is required for clinical management.

## Genome-Based Fungal Identification and Characterization

### DNA Metabarcoding for Precision Diagnosis of IFDs Directly From Clinical Specimens

The fundamental principle of DNA barcoding is that the intraspecies variation is less than the interspecies variation, effectively establishing a “barcoding gap.” An effort to standardize sequence-based identification of fungi led to the ribosomal ITS1/2 region being proposed as the primary fungal DNA barcode (Schoch et al., 2012). However, its universal use is compromised by the lack of quality controlled reference sequence databases. To overcome this problem, the ISHAM working group for Barcoding of Medical Fungi was established in 2010, which culminated in the establishment of the ISHAM ITS barcode database for human and animal pathogenic fungi^4^ in 2015 (Irinyi et al., 2015). Studying the intra- and interspecies variation of the ITS1/2 region in human pathogenic fungi confirmed earlier findings that this genetic locus was only able to identify approximately 75% of all fungal species accurately to the species level (Irinyi et al., 2016), prompting the search for a secondary fungal DNA barcode.

In a global study testing the genetic variability and ability to design universal primers for a variety of genetic loci, the translocation elongation factor 1 alpha (*TEF1α*) gene was proposed as the secondary fungal DNA barcode (Stielow et al., 2015). Again the lack of a quality-controlled reference database has hindered its widespread application in routine fungal diagnostics. Therefore, a complementary reference database for *TEF1α* was established in 2017 (seventh iBOL Conference, South Africa). The ISHAM Barcoding database, accessible at either www.its.mycologylab.org or www.isham.org (Meyer et al., 2019), includes quality controlled reference sequences for both ITS1/2 (*n* = 4200) and *TEF1α* (*n* = 2432) targets, representing 645 and 346 species, respectively, of human and animal pathogenic fungi. The Dual DNA barcoding system for fungi enables the identification of the majority of human and animal pathogenic fungi (Hoang et al., 2019).

The development of NGS has enabled the simultaneous sequencing of mixed microbial communities directly from a variety of clinical samples (e.g., blood, sputum, BALF, tissue, and stools) using either Illumina or Oxford Nanopore Technology (Lefterova et al., 2015; Zhou et al., 2016; Hong et al., 2018; Langelier et al., 2018; Blauwkamp et al., 2019; Charalampous et al., 2019; McTaggart et al., 2019). Sequences obtained from clinical samples through NGS can then be identified to the genus and species level by using sequence alignment tools such as BLAST or WIMP (Camacho et al., 2009; Juul et al., 2015) against appropriate publicly available quality-controlled reference sequence databases, e.g., ISHAM barcoding database UNITE, RefSeq, and BOLD (Hebert et al., 2003; Kõljalg et al., 2013; Schoch et al., 2014; Meyer et al., 2019). However, there are currently a number of major limitations in this technology which may lead to inaccurate or insufficient identification of the fungal pathogen, including: (i) pre-PCR biases, such as sample handling, contamination introduced during sample collection, aliquoting, nucleic acid extraction, library preparation, or pooling (Salter et al., 2014; Strong et al., 2014), DNA extraction methods including the choice of storage buffer and extraction kit (Hallmaier-Wacker et al., 2018), the quantity of host DNA, which could be reduced by using various depletion methods (Hasan et al., 2016), and the issues related to the extraction of DNA of adequate quality (high purity, high molecular weight, and high concentration) (Hasan et al., 2016; Hallmaier-Wacker et al., 2018; Sanderson et al., 2018; Nicholls et al., 2019); (ii) PCR biases, including primer mismatches and variable length of the obtained amplicon (Schloss and Westcott, 2011; Boers et al., 2019); (iii) high sequencing error rate of the current NGS technologies, especially long read sequencing (Bakker et al., 2012; Schirmer et al., 2015; Tyler et al., 2018); (iv) the lack of complete and quality-controlled reference sequence databases with correct taxonomic assignment (Irinyi et al., 2016; Greninger, 2018); and (v) lack of appropriate bioinformatic tools, including alignment algorithms and cross-talk between fungal sequences (Mulcahy-O’Grady and Workentine, 2016; Chiu and Miller, 2019). As such, any DNA metabarcoding-based pathogen identification should be interpreted and reviewed in the clinical context of the disease symptoms. With technological advancement and increasing moves to metagenomics approaches for clinical diagnostics, it is anticipated that these issues will be overcome. NGS-based DNA metabarcoding in combination with high quality reference sequence databases promises to drastically reduce the turnaround time for the diagnosis of IFDs to 24–48 h. But the main drawback of clinical metagenomics is currently the cost, running to approximately one million dollars at minimum after one considering the sequencing facility, computational infrastructure, and personnel required to run it (Greninger, 2018).

The introduction of culture and PCR free metagenomics for the direct detection of human pathogens in clinical specimens has to date only been applied to bacteria (Chiu and Miller, 2019) and has still to overcome major challenges associated with the low pathogen presence in a clinical sample, and the improvement of the DNA quality, sequencing library preparation methodology, computational bioinformatics pipelines, and the reduction of the associated sequencing costs. Our group was recently the first to report the use of MinION-based NGS sequencing to confirm the diagnosis of an IFI to detect *P. jirovecii* directly from BAL and sputum (Irinyi et al., 2019). Another group has developed an NGS-based method to detect a broad range of fungi in BAL specimens and applied it to the analysis of the fungal microbiome of the lung during fungal infection, demonstrating the potential to distinguish fungal infection from colonization (McTaggart et al., 2019).

### Whole Genome Sequencing for Epidemiological Studies, Outbreak Investigations, and Resistance Gene Detection

Next generation sequencing technologies enable the application of whole genome sequencing (WGS) to fungal genotyping without genetic insights or prior knowledge of the population structure of a species in question. As such, it can be used to investigate the genetic relatedness of isolates in an outbreak setting or for the detection of a specific mutation related to antifungal resistance. WGS is becoming the method of choice for molecular epidemiology studies, replacing traditional typing methods, including multi-locus sequence typing (MLST). WGS genotyping is based on the detection of single nucleotide polymorphisms (SNPs) through a genome-wide view which can be compared either in the presence of an already existing reference genome or after *de novo* genome assembly to enable genome-wide investigation.

Whole genome sequencing typing was applied to investigate a large healthcare related outbreak caused by *Exserohilum rostratum* meningitis in the United States following the use of methylprednisolone acetate (MPA) injections contaminated with this fungus. Isolates cultured from the MPA vials as well as clinical isolates were shown to be highly clonal, strongly indicating a single source. Two SNPs were identified among the outbreak-related isolates compared to hundreds of thousands of SNPs identified between the non-outbreak isolates (Litvintseva et al., 2014). Similarly, WGS SNP analysis was applied to the bloodstream infections caused by *Sarocladium kiliense* after administration of contaminated anti-nausea medication in oncology patients, with five SNPs being identified among the outbreak-related strains (Etienne et al., 2016). More recently, WGS typing was used to characterize four major clades of *C. auris* strains. Isolates within each clade were clonal, while there were thousands of SNPs between the clades. Additionally, WGS identified a specific mutation in the *ERG11* gene associated with azole resistance (Lockhart et al., 2017). Further examples of WGS applications in detection of resistance are discussed in Section “Molecular Detection of Antifungal Drug Resistance.”

Although the costs of NGS sequencing equipment, assembly, and analysis continue to fall, the lack of comprehensive reference genome sequence databases is a major challenge for the incorporation of WGS sequencing into routine infection control and outbreak investigations.

## Molecular Detection of Antifungal Drug Resistance

Antifungal susceptibility testing of fungal pathogens is a core function of the diagnostic mycology laboratory. Phenotypic methods have been standardized, and have established practice utility with interpretative minimum inhibitory concentration (MIC) clinical breakpoints (CBPs) in some *Candida* and *Aspergillus* species (Cuenca-Estrella, 2014). However, culture-based methods are slow, and may be hampered by poor growth rates (or for some molds, failure to sporulate), and a lack of interpretive criteria.

Molecular tools have good potential to offer more rapid results, and have the advantage of being able to determine the underlying genetic basis of resistance (Perlin and Wiederhold, 2017). However, many molecular methods are not standardized. Further, it is important that a particular genetic target can be linked to a validated mechanism of resistance for a particular drug. Ideally, the target should confer: (i) concordant results by MIC testing; (ii) an altered effector or downstream action following alteration of the drug target; and (iii) either documented resistance in animal models or clinical failure with treatment. Here we review and put into perspective, the role of molecular methods for assessing antifungal drug resistance. Although best illustrated with the echinocandins with *Candida* species and azoles with *A. fumigatus*, the *Candida*-azole “bug–drug” pair is also discussed.

### Molecular Methods to Detect Azole Resistance in *Candida* Species

Azole resistance in *Candida* species can arise through: (i) point mutations in various *Candida* genes; (ii) upregulation of drug efflux pumps or transporters; (iii) overexpression of the drug target; and (iv) cellular stress response factors (reviewed in Cuenca-Estrella, 2014; Cowen et al., 2015). The contribution of each of these mechanisms appears to vary by species but generally, upregulation and overexpression mechanisms play the greater role.

Point mutations in the *ERG11* gene, which encodes 14-alpha-demethylase, the target of the azoles, alters drug affinity and in *C. albicans* at least, also results in *ERG11* overexpression via mutations in the transcription factor *UPC2* (Flowers et al., 2012). Mutations have been best described in three “regional hotspots” of *ERG11* (amino acid positions 105–165, 266–287, and 405–488) and in the *ERG3* gene (T330A and A351V) (Martel et al., 2010), with again mutation studies best documented in *C. albicans*. These mutations can be reliably detected by allele-specific real-time molecular probes, DNA microarray technology, high-resolution melt curve analysis, and DNA sequence analysis (Perlin, 2009). These techniques are robust and can be used on primary specimens, but staff expertise is essential. Methods to detect multiple point mutations are best multiplexed, and if DNA sequencing is undertaken, the need for multiple sequencing runs is costly. Further, whether a detected mutation in the *ERG* gene family confers phenotypic resistance requires additional experiments to establish a causal effect as not all amino acid substitutions are linked to resistance (Jensen, 2016).

Instead, upregulation of the azole efflux pumps encoded by genes of the ATP binding cassette (ABC) or the major facilitator superfamilies (MFS) is the more important mechanism of resistance through changes in the regulation of their expression by mutations in the transcription factors, e.g., *TAC1* and *MRR1* (*C. albicans*) and *CgPDR1* (*C. glabrata*) (Vermitsky et al., 2006; Morschhäuser et al., 2007). *Candida* species also have plasticity in their genomes with movement within chromosomes, which can alter both efflux pump and *ERG11* expression (Perlin and Wiederhold, 2017). Finally, biofilm formation that limits drug access and impacts on resistance to the azoles and other drug classes is well described in *Candida* species, including the emerging *C. auris* (Sherry et al., 2017).

Because of the diversity of resistance mechanisms, pragmatic positioning of molecular methods to detect clinically relevant drug resistance is difficult. The exception may be in *C. glabrata* where azole resistance is associated with mutations in the transcription factor pdr1 (Ferrari et al., 2009), or if targeted DNA sequencing is performed for a mutation already known to confer resistance. Resistance mutations are broadly distributed throughout the genome and as such, NGS approaches have been studied for the detection of mutations in genes associated with azole resistance including in *ERG11*, *ERG3*, *TAC1*, and *CgPDR1* (Garnaud et al., 2015; Biswas et al., 2017; Castanheira et al., 2017). NGS further has potential to detect novel mutations implicated in phenotypic resistance that may otherwise be missed by targeted DNA sequencing. In *C. glabrata*, NGS has detected multiple SNPs in *CgPDR1* and *CgCDR1* in azole-resistant isolates; although none were definitively associated with resistance, azole-resistant isolates tended to have amino substitutions in pdr1 beyond the first 250 amino acid positions, unlike the susceptible isolates (Biswas et al., 2018). The cost of NGS is currently ∼AUD 80/sample (∼USD 57) but this is likely to fall with technological advances allowing ease of use for routine applications.

### Molecular Methods to Detect Azole Resistance in *Aspergillus fumigatus*

Overall, the utility of molecular methods for detecting azole resistance in *A. fumigatus* is more straightforward than in *Candida* species as there is a strong association between specific mutations in the *CYP51A* geneF (the homolog of *ERG11* of *Candida* species) and the azole-resistant phenotype, rendering this locus a well-suited target for a molecular-based assay. Amino acid substitutions in cyp51A are well described (Chowdhary et al., 2014) and in azole-resistant environmental isolates, mutations in the tandem base pair repeats of the promoter region of *CYP51A* coupled to amino acid substitutions in cyp51A leading to the following changes – TR_34_/L98H and TR_46_/Y121F/T289A (van der Linden et al., 2013). Other mechanisms of azole resistance include the overexpression of the *CYP51A*, and *ABC* and *MFS* genes as well as the gain of function mutation in the CCATT-binding transcription factor complex subunit hapE (Perlin and Wiederhold, 2017). However, the clinical relevance of these mechanisms is still uncertain.

To detect resistance-associated mutations, approaches such as real-time PCR, with or without molecular beacon probes, high-resolution melt curve analysis, DNA sequencing, and most recently NGS have all been used (Meletiadis et al., 2012; Hagiwara et al., 2014). The first real-time multiplex PCR assay was of an allele-specific molecular beacon design which differentiated wild type *CYP51A* from 7 mutant alleles at codon position 54 (Balashov et al., 2005). Other molecular beacon-based assays since including those employing TaqMan technology have shown similar good results, when validated against the gold standard of DNA sequencing. As such, the most commonly used is a conventional PCR amplifying the *CYP51A* gene and promoter regions with Sanger sequencing (Dudakova et al., 2017). Ahmad et al. (2014) developed a PCR-restriction fragment length polymorphism (PCR-RFLP) assay for detection of TR_34_ as well as the L98H substitution. However, NGS may be used to determine the genome-wide basis of resistance. Hagiwara et al. (2014) used NGS to identify non-synonymous mutations in sequential *A. fumigatus* isolates, which would have been missed by PCR-RFLP or microsatellite genotyping.

The above methods all require the availability of a cultured isolate for assessing resistance. Not uncommonly, IA is diagnosed by culture-independent methods; hence, azole resistance is probably under-reported. Several molecular assays that simultaneously detect *Aspergillus* spp. and azole resistance from clinical samples have been developed. They combine high sensitivity with high specificity to detect the small quantities of *Aspergillus* DNA in biological samples. Most formats are PCR-based followed by sequence analysis to identify mutations (Dudakova et al., 2017). Using a nested PCR, in culture-negative PCR-positive samples, the TR_34_/L98H and M220 mutations were detected in 55.1% of samples from patients with chronic pulmonary aspergillosis or ABPA (Denning et al., 2011).

The AsperGenius® and MycoGENIE commercial assays simultaneously detect *Aspergillus* spp. and common *CYP51A* mutations directly in clinical specimens (Table 1). AsperGenius® has performed well on BALF (including culture-negative fluid) and blood samples from hematology and ICU patients (Chong et al., 2015; White et al., 2017b). While the sensitivity, specificity, PPV, and NPV were 88.9, 89.3, 72.7, and 96.2%, respectively, for the hematology group, and 80.9, 93.3, 80, and 93.3%, respectively, for the ICU group, *CYP51A* mutations were detected in 2/14 positive *Aspergillus* PCRs from the combined patient groups (one TR_34_/L98H and one TR_46_/Y121F/T289A); the detected resistance mutations were associated with clinical failure of voriconazole therapy (Chong et al., 2015). The sensitivity and specificity of the MycoGENIE were 92.9 and 90.1%, respectively, using respiratory samples (*n* = 88) and 100 and 84.6%, respectively, with serum samples; this study detected no *CYP51A* mutants directly from specimen (Dannaoui et al., 2017). Another study compared the AsperGenius® and MycoGENIE assays for the detection of *Aspergillus* and resistance mutations in sputum specimens from cystic fibrosis patients (Guegan et al., 2018). Neither test detected any *CYP51A* resistance mutations, and the AsperGenius® had only limited success in amplifying the *CYP51A* gene targets, likely due to low fungal burden (Guegan et al., 2018). While time efficient, these commercial assays are limited by the fact that *CYP51A* is a single copy gene, and that they detect only azole resistance involving the tandem repeat. In Australia where the incidence of azole resistance is low (< 3%), 2/3 azole resistant isolates had the G54R mutation associated with high MICs to itraconazole and posaconazole (Talbot et al., 2018). A recent study from the United Kingdom employing the AsperGenius assay® (PathoNostics BV) showed that 16/22 (73%) isolates with the resistant phenotype harbored no mutations detected by this test (Abdolrasouli et al., 2018). The FungiplexR Aspergillus Azole-R IVD real-time PCR platform is also now available. It has two targets TR_34_ and TR_46_; clinical studies examining its utility for resistance detection are scarce. It should be noted, however, that since the *CYP51A* gene is single copy, and the target used for detection of *Aspergillus* is multi-copy, to date, the assays have greater utility in detecting *Aspergillus per se*, rather than detecting its resistance mutations. Expert opinion is that there is value in routinely evaluating only those samples with high fungal loads for azole resistance, and only in regions with a high prevalence of pan-azole resistance (Alanio and Bretagne, 2017). Further studies are required to determine whether the information provided by commercial assays leads to more rapid diagnosis of IA (Buchheidt et al., 2017).

### Molecular Methods to Detect Echinocandin Resistance in *Candida* spp.

Standard MIC measurement is at times, unable to reliably distinguish wildtype from non-wildtype isolates, particularly for caspofungin. The application of molecular methods offers good potential to rapidly detect echinocandin resistance which hinges upon detection of specific mutations in the *FKS* genes of *Candida*. *FKS* genotype analysis is a better predictor of resistance than MIC testing alone because the presence of *FKS* mutations correlates with high drug MICs and is an independent risk factor for therapeutic failure (summarized in Shields et al., 2012; Arendrup and Perlin, 2014).

Unlike the azoles, echinocandins are not impacted by the actions of multidrug efflux transporters and the resistant phenotype is well understood to be a result of a number of amino acid substitutions in “hot spot” regions of the Fks subunits (Arendrup and Perlin, 2014). In *Candida* species, there are three *FKS* genes – *FKS1*, *FKS2*, and *FKS3.* Mutations in *FKS1* alone will confer resistance in all species. In *C. glabrata*, mutations in *FKS2* will also confer resistant phenotypes (Arendrup and Perlin, 2014; Pham et al., 2014). Of the total number of ∼20 known mutations linked to resistance, only a few mutations account for 65–80% of observed echinocandin resistance (Perlin, 2015). In *C. albicans* for example, amino acid substitutions at positions Fks1p-S641 and Fks1p-S645 are the cause of nearly 90% of resistance seen. Molecular methods to date have only been evaluated using cultures and not directly on clinical specimens.

As such DNA sequencing lends itself as the logical method to resolve all known *FKS* mutations within 3–4 h. This requires knowledge of specific mutations that have been validated as resistance targets. Targeted gene sequencing of multiple genes is impractical and costly for clinical laboratories. Alternatives include: (i) real-time PCR assays with or without probe detection which can distinguish WT strains from those with *FKS* mutations (Balashov et al., 2006); (ii) microsphere-based assays using Luminex MagPix technology (Pham et al., 2014); and (iii) more sophisticated molecular beacon and melt curve assays (Zhao et al., 2016). A dual assay for *C. glabrata FKS1* and *FKS2* identified the predominant clinically relevant resistance-associated mutations in *FKS1* (e.g., leading to amino acid substitutions S629P, F625S) and *FKS2* (F659S, S663P) within 3 h (Zhao et al., 2016). A simple, rapid assay using classical PCR primer sets was developed to detect the 10 most common *FKS* mutations in *C. glabrata* within 4 h (Dudiuk et al., 2014).

Finally, NGS is suited to detecting a large number of mutations in multiple *FKS g*enes in clinical isolates of *C. glabrata* with high MICs to the echinocandins (Garnaud et al., 2015; Biswas et al., 2017). Biswas et al. (2017) used NGS to retrospectively study three strain pairs of *C. glabrata* from three patients where antifungal resistance developed during treatment. Two of three isolate pairs developed a > 60-fold increase in the MICs to all echinocandins and NGS detected mutations in either the *FKS1* (S629P) or *FKS2* (S663P) genes of the resistant isolates (Biswas et al., 2017).

## Issues Relating to Implementing New Technologies

Pathology (or diagnostic) stewardship is an area deserving consideration when implementing new technologies, and appropriate use of diagnostic tests for IFD should directly complement antifungal stewardship. As technical capabilities are being revolutionized by new technologies, there is a risk of exceeding laboratory capabilities to apply new tests effectively and efficiently; overuse of rapid diagnostic tests will increase healthcare costs without significantly improving healthcare outcomes. Therefore, consideration should be given to positioning particular tests to support specific patient groups or specimen types, taking in to consideration testing intervals, the significance of positive and negative results, and how results can be communicated with clinicians in a timely manner (Messacar et al., 2017).

From the laboratory perspective, when new tests are implemented the validation, obtaining sufficient “positive control” material, and other technical issues such as fungal contamination of commercial reagents (Harrison et al., 2010) need to be considered. Consideration must be given to how the results of molecular tests will be interpreted, e.g., as infection, colonization, or contamination. We are not yet in a position for molecular technologies to replace microscopy and culture for diagnosis of IFD, or broth microdilution antifungal susceptibility testing, although microbiologists should be prepared to transition to these methods when the time arrives.

Cost-effectiveness is another critical factor to be considered before implementing any new diagnostic method. Additionally, while outsourcing low throughput “boutique” pathology tests is common practice to reduce costs, this increases the turnaround time, raising the possibility that the test results may not be available in a suitable timeframe to impact patient management. Therefore, consideration must be given to implementing fungal diagnostic tests that are easily accessible. Using the example of NGS, sequencing costs are decreasing, and therefore likely to become a viable option for many pathology laboratories in the near future. Cost considerations will be the initial laboratory setup, cost of the instrumentation, and training laboratory staff. In the longer term, costs would include reagents and accessories, and equipment servicing. If the NGS service is not in-house, costs may be higher and turnaround times longer. Implementing IVD molecular tests may not be as costly if the laboratory already has access to a real-time PCR platform; in this case, the bulk of ongoing costs are likely to be related to kit procurement; and while costs may exceed those of culture and microscopy, considering the overall cost of healthcare rather than the immediate cost to pathology laboratories may see a cost benefit. An Australian study examined the cost–benefit of a biomarker-based diagnostic strategy using data from a randomized control trial of IA in HSCT patients and individual patient costings (hospital length of stay, treatments costs, pathology test costs); costs were determined for 137 proven or probable IA patients at four Australian centers. While the biomarker-based diagnostic strategy was not inexpensive, it was found to be cost-effective if a survival benefit is maintained over several years (Macesic et al., 2017); the cost benefit in this case would have to be considered over the scope of the overall health budget, rather than just that of the pathology provider. The cost-effectiveness of a *Candida* PCR test was examined in terms of the use of fluconazole versus the more expensive echinocandin drugs as empiric treatment of *Candida* peritonitis, when traditionally fluconazole resistant species (e.g., *C. glabrata*, *C. krusei*) could be quickly detected by the PCR. This study found that use of fluconazole empirical treatment with PCR to detect the fluconazole resistant species (changing over to an echinocandin) is more cost effective than using fluconazole empirical treatment without PCR (Pagès et al., 2017). Furthermore, a recent analysis of the cost-effectiveness of T2Candida determined it was less costly than blood culture-directed and empiric echinocandin therapy. Patch et al. (2018) found the mean duration of empiric treatment in patients with T2Candida-negative/BC-negative results was 2.4 days, compared with 6.7 days prior to the introduction of T2Candida testing. The estimated financial savings associated with reduced use of empiric therapy was US$280 per patient.

## Concluding Remarks

Advances in molecular diagnostic technologies have undoubtedly improved the landscape for fungal diagnostics and identification, enabling a range of tests for diagnosis and/or screening at risk patients for IFDs, with rapid turnaround times, and covering a broad range of IFDs. Increasing experience with PCR assays to directly detect fungi in clinical specimens with clinical validation studies has positioned these assay types well on the way to becoming routine in clinical laboratories. Similarly, standardization of *Aspergillus* PCR enables robust inter-laboratory comparisons and has been included as a biomarker for IA in clinical trials. Molecular assays that directly detect *Candida* spp. from blood cultures or WB have good potential to be used in conjunction with other fungal biomarkers to inform the likelihood of infection. It is envisaged that as other genera/species-specific assays advance toward standardization, they will also have a greater role to play in routine diagnostics, as will molecular assays that enable simultaneous detection of pathogens and their major resistance markers. Finally, with high-level DNA barcoding, NGS technologies, and metagenomic approaches, the vision of a “one stop shop” for fungal biomarkers seems to be within reach in the foreseeable future. This wealth of data will require parallel studies to examine their clinical applications.

## Author Contributions

SK, SC, and CH contributed equally to developing the concept and writing and editing the manuscript. WM contributed to writing and editing the manuscript.

## Conflict of Interest

The authors declare that the research was conducted in the absence of any commercial or financial relationships that could be construed as a potential conflict of interest.

## Footnotes

1 www.fpcri.eu

2 www.eapcri.eu; www.fpcri.eu

3 https://www.clinicaltrials.gov/ct2/show/NCT02845934

4 http://its.mycologylab.org/

## References

[B1] Abdolrasouli A. Scourfield A. Rhodes J. Shah A. Elborn J. S. Fisher M. C. (2018). High prevalence of triazole resistance in clinical *Aspergillus fumigatus* isolates in a specialist cardiothoracic centre. *Int. J. Antimicrob. Agents* 52 637–642. 10.1016/j.ijantimicag.2018.08.004 30103005

[B2] Ahmad S. Khan Z. Hagen F. Meis J. F. (2014). Simple, low-cost molecular assays for TR34/L98H mutations in the *cyp51A* gene for rapid detection of triazole-resistant *Aspergillus fumigatus* isolates. *J. Clin. Microbiol.* 52 2223–2227. 10.1128/JCM.00408-14 24719446 PMC4042779

[B3] Ala-Houhala M. Koukila-Kähkölä P. Antikainen J. Valve J. Kirveskari J. Anttila V. J. (2018). Clinical use of fungal PCR from deep tissue samples in the diagnosis of invasive fungal diseases: a retrospective observational study. *Clin. Microbiol. Infect.* 24 301–305. 10.1016/j.cmi.2017.08.017 28870728

[B4] Alanio A. Bretagne S. (2017). Performance evaluation of multiplex PCR including *Aspergillus* – not so simple! *Med. Mycol*. 55 56–62. 10.1093/mmy/myw080 27664168

[B5] Alanio A. Desoubeaux G. Sarfati C. Hamane S. Bergeron A. Azoulay E. (2011). Real-time PCR assay-based strategy for differentiation between active *Pneumocystis jirovecii* pneumonia and colonization in immunocompromised patients. *Clin. Microbiol. Infect.* 17 1531–1537. 10.1111/j.1469-0691.2010.03400.x 20946413

[B6] Alanio A. Hauser P. M. Lagrou K. Melchers W. J. Helweg-Larsen J. Matos O. (2016). ECIL guidelines for the diagnosis of *Pneumocystis jirovecii* pneumonia in patients with haematological malignancies and stem cell transplant recipients. *J. Antimicrob. Chemother.* 71 2386–2396. 10.1093/jac/dkw156 27550991

[B7] Alvarez-Martínez M. J. Miró J. M. Valls M. E. Mas J. de la Bellacasa J. P. Sued O. (2010). Prevalence of dihydropteroate synthase genotypes before and after the introduction of combined antiretroviral therapy and their influence on the outcome of Pneumocystis pneumonia in HIV-1-infected patients. *Diagn. Microbiol. Infect. Dis.* 68 60–65. 10.1016/j.diagmicrobio.2010.04.007 20727472

[B8] Arendrup M. C. Perlin D. S. (2014). Echinocandin resistance: an emerging clinical problem? *Curr. Opin. Infect. Dis*. 27 484–492. 10.1097/QCO.0000000000000111 25304391 PMC4221099

[B9] Arvanitis M. Anagnostou T. Fuchs B. B. Caliendo A. M. Mylonakis E. (2014). Molecular and non-molecular diagnostic methods for invasive fungal infections. *Clin. Microbiol. Rev.* 27 490–526. 10.1128/cmr.00091-13 24982319 PMC4135902

[B10] Arvanitis M. Mylonakis E. (2015). Diagnosis of invasive aspergillosis: recent developments and ongoing challenges. *Eur. J. Clin. Invest.* 45 646–652. 10.1111/eci.12448 25851301

[B11] Avni T. Leibovici L. Paul M. (2011). PCR diagnosis of invasive candidiasis: systematic review and meta-analysis. *J. Clin. Microbiol.* 49 665–670. 10.1128/JCM.01602-10 21106797 PMC3043518

[B12] Avni T. Levy I. Sprecher H. Yahav D. Leibovici L. Paul M. (2012). Diagnostic accuracy of PCR alone compared to galactomannan in bronchoalveolar lavage fluid for diagnosis of invasive pulmonary aspergillosis: a systematic review. *J. Clin. Microbiol.* 50 3652–3658. 10.1128/JCM.00942-12 22952268 PMC3486225

[B13] Bakker M. G. Tu Z. J. Bradeen J. M. Kinkel L. L. (2012). Implications of pyrosequencing error correction for biological data interpretation. *PLoS One* 7:e44357. 10.1371/journal.pone.0044357 22952965 PMC3431371

[B14] Balashov S. V. Gardiner R. Park S. Perlin D. S. (2005). Rapid high-throughput mulitplex, real-time PCR for identificaion of mutations in the c*yp51A* gene of *Aspergillus fumigatus* that confer resistance to itraconazole. *J. Clin. Microbiol.* 43 214–222. 10.1128/jcm.43.1.214-222.2005 15634974 PMC540178

[B15] Balashov S. V. Park S. Perlin D. S. (2006). Assessing reisstance to the echinocanind anitfingal drug caspofungin in *Candida albicans* by profiling mutations in *FKS1*. *Antimicrob. Agents Chemother.* 50 2058–2063. 10.1128/aac.01653-05 16723566 PMC1479158

[B16] Baldin C. Soliman S. S. M. Jeon H. H. Alkhazraji S. Gebremariam T. Gu Y. (2018). PCR-based approach targeting Mucorales-specific gene family for diagnosis of mucormycosis. *J. Clin. Microbiol.* 56:e746-18. 10.1128/JCM.00746-18 30068535 PMC6156309

[B17] Barnes R. A. White P. L. Morton C. O. Rogers T. R. Cruciani M. Loeffler J. (2018). Diagnosis of aspergillosis by PCR: clinical considerations and technical tips. *Med. Mycol.* 56 S60–S72. 10.1093/mmy/myx091 29087518

[B18] Bezdicek M. Lengerova M. Ricna D. Weinbergerova B. Kocmanova I. Volfova P. (2016). Rapid detection of fungal pathogens in bronchoalveolar lavage samples using panfungal PCR combined with high resolution melting analysis. *Med. Mycol.* 54 714–724. 10.1093/mmy/myw032 27161789

[B19] Biswas C. Chen S. C. Halliday C. Kennedy K. Playford E. G. Marriott D. J. (2017). Identification of genetic markers of resistance to echinocandins, azoles and 5-fluorocytosine in *Candida glabrata* by next-generation sequencing: a feasibility study. *Clin. Microbiol. Infect.* 23 676.e7–676.e10. 10.1016/j.cmi.2017.03.014 28344162

[B20] Biswas C. Marcelino V. van Hal S. Halliday C. Martinez E. Wang Q. (2018). Whole genome sequencing of Australian *Candida glabrata* isolates revelas genetic diverty and novel sequence tyes. *Front. Microbiol.* 9:2946. 10.3389/fmicb.2018.02946 30559734 PMC6287553

[B21] Blauwkamp T. A. Thair S. Rosen M. J. Blair L. Lindner M. S. Vilfan I. D. (2019). Analytical and clinical validation of a microbial cell-free DNA sequencing test for infectious disease. *Nat. Microbiol.* 4 663–674. 10.1038/s41564-018-0349-6 30742071

[B22] Boers S. A. Jansen R. Hays J. P. (2019). Understanding and overcoming the pitfalls and biases of next-generation sequencing (NGS) methods for use in the routine clinical microbiological diagnostic laboratory. *Eur. J. Clin. Microbiol. Infect. Dis.* 38 1059–1070. 10.1007/s10096-019-03520-3 30834996 PMC6520317

[B23] Bousbia S. Papazian L. Saux P. Forel J. M. Auffray J. P. Martin C. (2012). Repertoire of intensive care unit pneumonia microbiota. *PLoS One* 7:e32486. 10.1371/journal.pone.0032486 22389704 PMC3289664

[B24] Brown G. D. Denning D. W. Gow N. A. Levitz S. M. Netea M. G. White T. C. (2012). Hidden killers: human fungal infections. *Sci. Transl. Med.* 4:165rv13. 10.1126/scitranslmed.3004404 23253612

[B25] Buchheidt D. Reinwald M. Hofmann W. K. Boch T. Spiess B. (2017). Evaluating the use of PCR for diagnosing invasive aspergillosis. *Expert Rev. Mol. Diagn.* 17 603–610. 10.1080/14737159.2017.1325735 28460550

[B26] Caillot D. Valot S. Lafon I. Basmaciyan L. Chretien M. L. Sautour M. (2016). Is it time to include CT “reverse halo sign” and qPCR targeting Mucorales in serum to EORTC-MSG criteria for the diagnosis of pulmonary mucormycosis in leukemia patients? *Open Forum Infect. Dis*. 3:ofw190. 10.1093/ofid/ofw190 28101518 PMC5225907

[B27] Camacho C. Coulouris G. Avagyan V. Ma N. Papadopoulos J. Bealer K. (2009). BLAST+: architecture and applications. *BMC Bioinformatics* 10:421. 10.1186/1471-2105-10-421 20003500 PMC2803857

[B28] Castanheira M. Deshpande L. M. Davis A. P. Rhomberg P. R. Pfaller M. A. (2017). Monitoring antifungal resistance in a global collection of invasive yeasts and molds: application of CLSI epidemiological cutoff values and whole-genome sequencing analysis for detection of azole resistance in *Candida albicans*. *Antimicrob. Agents Chemother.* 61 e906–e917. 10.1128/AAC.00906-17 28784671 PMC5610521

[B29] Chapman B. Slavin M. Marriott D. Halliday C. Kidd S. Arthur I. (2017). Changing epidemiology of candidaemia in Australia. *J. Antimicrob. Chemother.* 72 1103–1108. 10.1093/jac/dkw422 28364558

[B30] Charalampous T. Kay G. L. Richardson H. Aydin A. Baldan R. Jeanes C. (2019). Nanopore metagenomics enables rapid clinical diagnosis of bacterial lower respiratory infection. *Nat. Biotechnol.* 37 783–792. 10.1038/s41587-019-0156-5 31235920

[B31] Charnot-Katsikas A. Tesic V. Love N. Hill B. Bethel C. Boonlayangoor S. (2017). Use of the accelerate pheno system for identification and antimicrobial susceptibility testing of pathogens in positive blood cultures and impact on time to results and workflow. *J. Clin. Microbiol.* 56:e1166-17. 10.1128/JCM.01166-17 29118168 PMC5744213

[B32] Chiu C. Y. Miller S. A. (2019). Clinical metagenomics. *Nat. Rev Genet.* 20 341–355. 30918369 10.1038/s41576-019-0113-7 PMC6858796

[B33] Chong G. L. van de Sande W. W. Dingemans G. J. Gaajetaan G. R. Vonk A. G. Hayette M. P. (2015). Validation of a new *Aspergillus* real-time PCR assay for direct detection of *Aspergillus* and azole resistance of *Aspergillus fumigatus* on bronchoalveolar lavage fluid. *J. Clin. Microbiol.* 53 868–874. 10.1128/JCM.03216-14 25568431 PMC4390672

[B34] Chowdhary A. Sharma C. Hagen F. Meis J. F. (2014). Exploring azole antifungal drug resistance in *Aspergillus fumigatus* with special reference to resistance mechanisms. *Future Microbiol.* 9 9697–9711. 10.2217/fmb.14.27 24957095

[B35] Clancy C. J. Nguyen M. H. (2013). Finding the “missing 50%” of invasive candidiasis: how nonculture diagnostics will improve understanding of disease spectrum and transform patient care. *Clin. Infect. Dis.* 56 1284–1292. 10.1093/cid/cit006 23315320

[B36] Clancy C. J. Nguyen M. H. (2018a). Diagnosing invasive candidiasis. *J. Clin. Microbiol.* 56 e1909–e1917. 10.1128/JCM.01909-17 PMC5925725 29444828

[B37] Clancy C. J. Nguyen M. H. (2018b). Non-culture diagnostics for invasive candidiasis: promise and unintended consequences. *J. Fungi* 4:E27. 10.3390/jof4010027 29463043 PMC5872330

[B38] Clancy C. J. Nguyen M. H. (2018c). T2 magnetic resonance for the diagnosis of bloodstream infections: charting a path forward. *J. Antimicrob. Chemother.* 73(Suppl. 4), iv2–iv5. 10.1093/jac/dky050 29608754

[B39] Clancy C. J. Pappas P. G. Vazquez J. Judson M. A. Kontoyiannis D. P. Thompson G. R. III (2018). Detecting infections rapidly and easily for candidemia trial, part 2 (DIRECT2): a prospective, multicentre study of the T2Candida panel. *Clin. Infect. Dis.* 66 1678–1686. 10.1093/cid/cix1095 29438475

[B40] Cornerly O. A. Arikan-Akdagli S. Dannaoui E. Groll A. H. Lagrou K. Chakrabarti A. (2014). ESCMID and ECMM joint clinical guidelines for the diagnosis and management of mucormycosis 2013. *Clin. Microbiol. Infect.* 20 5–25. 10.1111/1469-0691.12371 24479848

[B41] Cowen L. E. Sanglard D. Howard S. J. Rogers P. D. Perlin D. S. (2015). Mechanisms of antifungal drug resistance. *Cold Spring Harb. Perspect. Med.* 5:a019752. 10.1101/cshperspect.a019752 25384768 PMC4484955

[B42] Cruciani M. Mengoli C. Loeffler J. Donnelly J. P. Loeffler J. Jones B. L. (2015). Polymerase chain reaction blood tests for the diagnosis of invasive aspergillosis in immunocompromised people. *Cochrane Database Syst. Rev.* 7:CD009551. 10.1002/14651858.CD009551.pub2 26343815

[B43] Cuenca-Estrella M. (2014). Antifungal drug resistance mechanisms in pathogenic fungi: from bench to bedside. *Clin. Microbiol. Infect.* 20(Suppl. 6), 54–59. 10.1111/1469-0691.12495 24372680

[B44] Dannaoui E. Gabriel F. Gaboyard M. Lagardere G. Audebert L. Quesne G. (2017). Molecular diagnosis of invasive aspergillosis and detection of azole resistance by a newly commercialized PCR Kit. *J. Clin. Microbiol.* 55 3210–3218. 10.1128/JCM.01032-17 28814586 PMC5654904

[B45] Danylo A. Courtemanche C. Pelletier R. Boudreault A. A. (2014). Performance of MycAssay *Aspergillus* real-time assay compared with the galactomannan detection assay for the diagnosis of invasive aspergillosis from serum samples. *Med. Mycol.* 52 577–583. 10.1093/mmy/myu025 25023484

[B46] Dellière S. Rivero-Menendez O. Gautier C. Garcia-Hermoso D. Alastrey-Izquierdo A. Alanio A. (2019). Emerging mould infections: get prepared to meet unexpected fungi in your patient. *Med. Mycol.* [Epub ahead of print] 10.1093/mmy/myz039 31111906

[B47] Denning D. K. Park S. Lass-Flörl C. Fraczek M. G. Kirwan M. Gore R. (2011). High frequency triazole resistance in nonculturable *Aspergillus fumigatus* from lungs of patients with chronic fungal disease. *Clin. Infect. Dis.* 52 1123–1129. 10.1093/cid/cir179 21467016 PMC3106268

[B48] Desoubeaux G. Bailly É Guillaume C. De Kyvon MA Tellier A. C. Morange V. (2018). *Candida auris* in contemporary mycology labs: a few practical tricks to identify it reliably according to one recent French experience. *J. Mycol. Med*. 28 407–410. 10.1016/j.mycmed.2018.02.011 29567284

[B49] Desoubeaux G. Franck-Martel C. Caille A. Drillaud N. Lestrade Carluer de Kyvon M. A. Bailly É, et al. (2017). Use of calcofluor-blue brightener for the diagnosis of Pneumocystis jirovecii pneumonia in bronchial-alveolar lavage fluids: a single-center prospective study. *Med. Mycol.* 55 295–301. 10.1093/mmy/myw068 27562860

[B50] Donnelly J. P. Chen S. C. Kauffman C. A. Steinbach W. J. Baddley J. W. Verweij P. E. (2019). Revision and update of the consensus definitions of invasive fungal disease from the European Organization for Research and Treatment of Cancer and the Mycoses Study Group Education and Research Consortium. *Clin. Infect. Dis.* [Epub ahead of print] 31802125 10.1093/cid/ciz1008 PMC7486838

[B51] Dudakova A. Spiess B. Tangwattanachuleeporn M. Sasse C. Buchheidt D. Weig M. (2017). Molecular tools for the detection and deduction of azole antifungal drug resistance phenotypes in *Aspergillus* species. *Clin. Microbiol. Rev.* 30 1065–1091. 10.1128/CMR.00095-16 28903985 PMC5608879

[B52] Dudiuk C. Gamarra S. Leonardeli F. Jimenez-Ortigosa C. Vitale R. G. Afeltra J. (2014). Set of classical PCRs for detection of mutations in *Candida glabrata FKS* genes linked with echinocandin resistance. *J. Clin. Microbiol.* 58 4690–4696. 10.1128/JCM.01038-14 24829248 PMC4097693

[B53] Etienne K. A. Roe C. C. Smith R. M. Vallabhaneni S. Duarte C. Escandon P. (2016). Whole-genome sequencing to determine origin of multinational outbreak of *Sarocladium kiliense* bloodstream infections. *Emerg. Infect. Dis.* 22 476–481. 10.3201/eid2203.151193 26891230 PMC4766898

[B54] Fan L. C. Lu H. W. Cheng K. B. Li H. P. Xu J. F. (2013). Evaluation of PCR in bronchoalveolar lavage fluid for diagnosis of *Pneumocystis jirovecii* pneumonia: a bi-variate meta-analysis and systematic review. *PLoS One* 8:e73099. 10.1371/journal.pone.0073099 24023814 PMC3762835

[B55] Fauchier T. Hasseine L. Gari-Toussaint M. Casanova V. Marty P. M. Pomares C. (2016). Detection of *Pneumocystis jirovecii* by quantitative PCR to differentiate colonisation and pneumonia in immunocompromised HIV-positive and HIV-negative patients. *J. Clin. Microbiol.* 54 1487–1495. 10.1128/jcm.03174-15 27008872 PMC4879311

[B56] Ferrari S. Ischer F. Calabrese D. Posteraro B. Sanguinetti M. Fadda G. (2009). Gain of function mutations in CgPDR1 of *Candida glabrata* not only mediate antifungal resistance but also enhance virulence. *PLoS Pathog.* 5:e1000268. 10.1371/journal.ppat.1000268 19148266 PMC2607542

[B57] Flowers S. A. Barker K. S. Berkow E. L. Toner G. Chadwick S. G. Gygax S. E. (2012). Gain-of-function mutations in UPC2 are a frequent cause of *ERG11* upregulation in azole-resistant clinical isolates of *Candida albicans*. *Eukarot. Cell* 11 1289–1299. 10.1128/EC.00215-12 22923048 PMC3485914

[B58] Fortún J. Martin-Davila P. Gomez-Garcia de la Pedrosa E. Pintado V. Cobo J. Fresco G. (2012). Emerging trends in candidaemia: a higher incidence but similar outcome. *J. Infect.* 65 64–70. 10.1016/j.jinf.2012.02.011 22369861

[B59] Fortún J. Meije Y. Buitrago M. J. Gago S. Bernal-Martinez L. Pemán J. (2014). Clinical validation of a multiplex real-time assay for detection of invasive candidiasis in intensive care unit patients. *J. Antimicrob. Chemother.* 69 3134–3141. 10.1093/jac/dku225 24970743

[B60] Gaajetaan G. van Tegelen D. Kampermann T. Dingemans G. (2018). “Development of the first commercial real-time PCR assay for detection of Mucorales species,” in *Proceedings of the 20th Congress of the International Society for Human and Animal Mycology (ISHAM)*, Amsterdam.

[B61] Garnaud C. Botterel F. Sertour N. Bougnoux M. E. Dannaoui E. Larrat S. (2015). Next-generation sequencing offers new insights into the resistance of *Candida* spp. to echinocandins and azoles. *J. Antimicrob. Chemother.* 70 2556–2565. 10.1093/jac/dkv139 26017039

[B62] Gholinejad-Ghadi N. Shokohi T. Seifi Z. Aghili S. R. Roilides E. Nikkhah M. (2018). Identification of Mucorales in patients with proven invasive mucormycosis by polymerase chain reaction in tissue samples. *Mycoses* 61 909–915. 10.1111/myc.12837 30091261

[B63] Gits-Muselli M. White P. L. Mengoli C. Chen S. Crowley B. Dingemans G. (2019). The Fungal PCR Initiative’s evaluation of in-house and commercial *Pneumocystis jirovecii* qPCR assays: towards a standard for a diagnostic assay. *Med. Mycol.* [Epub ahead of print] 10.1093/mmy/myz115 31758173

[B64] Gomez C. A. Budvytiene I. Zemek A. J. Banaei N. (2017). Performance of targeted fungal sequencing for culture-independent diagnosis of invasive fungal disease. *Clin. Infect. Dis.* 65 2035–2041. 10.1093/cid/cix728 29020284

[B65] Greninger A. L. (2018). The challenge of diagnostic metagenomics. *Expert Rev. Mol. Diagn.* 18 605–615. 10.1080/14737159.2018.1487292 29898605

[B66] Guegan H. Chevrier S. Belleguic C. Deneuville E. Robert-Gangneux F. Gangneux J. P. (2018). Performance of molecular approaches for *Aspergillus* detection and azole resistance surveillance in cystic fibrosis. *Front. Microbiol.* 9:531 10.3389/fmicb.2018.00531 PMC5880881 29636731

[B67] Hagiwara D. Takahashi H. Watanabe A. Takahashi-Nakaguchi A. Kawamoto S. Kamei K. (2014). Whole-genome comparison of *Aspergillus fumigatus* strains serially isolated from patients with aspergillosis. *J. Clin. Microbiol.* 52 4202–4209. 10.1128/JCM.01105-14 25232160 PMC4313286

[B68] Halliday C. L. Kidd S. E. Sorrell T. C. Chen S. C.-A. (2015). Molecular diagnostic methods for invasive fungal disease: the horizon draws nearer? *Pathology* 47 257–269. 10.1097/PAT.0000000000000234 25719852

[B69] Hallmaier-Wacker L. K. Lueert S. Roos C. Knauf S. (2018). The impact of storage buffer. DNA extraction method, and polymerase on microbial analysis. *Sci. Rep.* 8:6292. 10.1038/s41598-018-24573-y 29674641 PMC5908915

[B70] Hammond S. P. Bialek R. Milner D. A. Petschnigg E. M. Baden L. R. Marty F. M. (2011). Molecular methods to improve diagnosis and identification of mucormycosis. *J. Clin. Microbiol.* 49 2151–2153. 10.1128/JCM.00256-11 21508149 PMC3122746

[B71] Harrison E. Stalhberger T. Whelan R. Sugrue M. Wingard J. R. Alexander B. D. (2010). *Aspergillus* DNA contamination in blood collection tubes. *Diagn. Microbiol. Infect. Dis.* 67 392–394. 10.1016/j.diagmicrobio.2010.02.028 20638611 PMC2907359

[B72] Hasan M. R. Rawat A. Tang P. Jithesh P. V. Thomas E. Tan R. (2016). Depletion of human DNA in spiked clinical specimens for improvement of sensitivity of pathogen detection by next-generation sequencing. *J. Clin. Microbiol.* 54 919–927. 10.1128/JCM.03050-15 26763966 PMC4809942

[B73] Hebert P. D. Cywinska A. Ball S. L. deWaard J. R. (2003). Biological identifications through DNA barcodes. *Philos. Trans. R. Soc. Lond. B Biol. Sci.* 270 313–321. 10.1098/rspb.2002.2218 12614582 PMC1691236

[B74] Heng S. C. Chen S. C. Morrissey C. O. Thursky K. Manser R. L. De Silva H. D. (2014). Clinical utility of *Aspergillus* galactomannan and PCR in bronchoalveolar lavage fluid for the diagnosis of invasive aspergillosis in patients with haematological malignancies. *Diagn. Microbiol. Infect.* 79 322–327. 10.1016/j.diagmicrobio.2014.03.020 24768294

[B75] Herrera S. Pavone P. Kumar D. Singer L. Humar A. Chaparro C. (2019). Chronic *Candida dubliniensis* meningitis in a lung transplant recipient. *Med. Mycol. Case Rep.* 24 41–43. 10.1016/j.mmcr.2019.03.004 30976504 PMC6439223

[B76] Hoang M. T. V. Irinyi L. Chen S. C. A. Sorrell T. C. Isham Barcoding for Medical Fungi Working Group Meyer W. (2019). Dual DNA barcoding for the molecular identification of the agents of invasive fungal infections. *Front. Microbiol.* 10:1647. 10.3389/fmicb.2019.01647 31379792 PMC6657352

[B77] Hong D. K. Blauwkamp T. A. Kertesz M. Bercovici S. Truong C. Banaei N. (2018). Liquid biopsy for infectious diseases: sequencing of cell-free plasma to detect pathogen DNA in patients with invasive fungal disease. *Diagn. Microbiol. Infect. Dis.* 92 210–213. 10.1016/j.diagmicrobio.2018.06.009 30017314

[B78] Huang L. Crothers K. Atzori C. Benfield T. Miller R. Rabodonirina M. (2004). Dihydropteroate synthase gene mutations in Pneumocystis and sulfa resistance. *Emerg. Infect. Dis.* 10 1721–1728. 10.3201/eid1010.030994 15504256 PMC3323267

[B79] Huang T. D. Melnik E. Bogaerts P. Evrard S. Glupczynski Y. (2019). Evaluation of the ePlex Blood Culture identification panels for detection of pathogens in bloodstream infections. *J. Clin. Microbiol.* 57:e1597-18. 10.1128/JCM.01597-18 30487304 PMC6355516

[B80] Huh H. J. Lim K. R. Ki C.-S. Huh K. Shim H. J. Song D. J. (2019). Comparative evaluation between the RealStar *Pneumocystis jirovecii* PCR kit and the AmpliSens *Pneumocystis jirovecii* (carinii)-FRT PCR kit for detecting *P. jirovecii* in non-HIV immunocompromised patients. *Ann. Lab. Med.* 39 176–182. 10.3343/alm.2019.39.2.176 30430780 PMC6240529

[B81] Imbert S. Meyer I. Palous M. Brossas J. Y. Uzunov M. Touafek F. (2018). *Aspergillus* PCR in bronchoalveolar lavage fluid for the diagnosis and prognosis of aspergillosis in patients with hematological and non-hematological conditions. *Front. Microbiol.* 9:1877. 10.3389/fmicb.2018.01877 30154779 PMC6102318

[B82] Irinyi L. Hu Y. Hoang T. V. M. Pasic L. Halliday C. Jayawardena M. (2019). Long-read sequencing based clinical metagenomics for the detection and confirmation of *Pneumocystis jirovecii* directly from clinical specimens – a paradigm shift in mycological diagnostics. *Med. Mycol.* [Epub ahead of print] 31758176 10.1093/mmy/myz109

[B83] Irinyi L. Lackner M. de Hoog S. Meyer W. (2016). DNA barcoding of fungi causing infections in humans and animals. *Fung Biol.* 120 125–136. 10.1016/j.funbio.2015.04.007 26781368

[B84] Irinyi L. Serena C. Garcia-Hermoso D. Arabatzis M. Desnos-Ollivier M. Vu D. (2015). International society of human and animal mycology (ISHAM)-ITS reference DNA barcoding database-the quality controlled standard tool for routine identification of human and animal pathogenic fungi. *Med. Mycol*. 53 313–337. 10.1093/mmy/myv008 25802363

[B85] Jensen R. H. (2016). Resistance in human pathogenic yeasts and filamentous fungi: prevalence, underlying molecular mechanisms and link to the use of antifungals in humans and the environment. *Dan, Med, J.* 63:B5288. 27697142

[B86] Juul S. Izquierdo F. Hurst A. Dai X. Wright A. Kulesha E. (2015). What’s in my pot? Real-time species identification on the MinION. *bioRxiv* [Preprint]

[B87] Kõljalg U. Nilsson R. H. Abarenkov K. Tedersoo L. Taylor A. F. Bahram M. (2013). Towards a unified paradigm for sequence-based identification of fungi. *Mol. Ecol.* 22 5271–5277. 10.1111/mec.12481 24112409

[B88] Komatsu H. Fujisawa T. Inui A. Horiuchi K. Hashizume H. Sogo T. (2004). Molecular diagnosis of cerebral aspergillosis by sequence analysis with panfungal polymerase chain reaction. *J. Pediatr. Hematol. Oncol.* 26 40–44. 10.1097/00043426-200401000-00013 14707712

[B89] Lackner M. Caramalho R. Lass-Florl C. (2014). Laboratory diagnosis of mucormycosis: current status and future perspectives. *Future Microbiol.* 9 683–695. 10.2217/fmb.14.23 24957094

[B90] Landlinger C. Preuner S. Bašková L. van Grotel M. Hartwig N. G. Dworzak M. (2010). Diagnosis of invasive fungal infections by a real-time pan fungal PCR assay in immunocompromised pediatric patients. *Leukemia* 24 2032–2038. 10.1038/leu.2010.209 20882044

[B91] Langelier C. Kalantar K. L. Moazed F. Wilson M. R. Crawford E. D. Deiss T. (2018). Integrating host response and unbiased microbe detection for lower respiratory tract infection diagnosis in critically ill adults. *Proc. Natl. Acad. Sci. U.S.A.* 115 e12353–e12362. 10.1073/pnas.1809700115 30482864 PMC6310811

[B92] Lau A. Chen S. Sorrell T. Carter D. Malik R. Martin P. (2007). Development and clinical application of a panfungal PCR assay to detect and identify fungal DNA in tissue specimens. *J. Clin. Microbiol.* 45 380–385. 10.1128/jcm.01862-06 17122000 PMC1829013

[B93] Lee S. H. Chen S. Y. Chien J. Y. Lee T. F. Chen J. M. Hsueh P. R. (2019). Usefulness of the FilmArray meningitis/encephalitis (M/E) panel for the diagnosis of infectious meningitis and encephalitis in Taiwan. *J. Microbiol. Immunol. Infect.* 52 760–768. 10.1016/j.jmii.2019.04.005 31085115

[B94] Lefterova M. I. Suarez C. J. Banaei N. Pinsky B. A. (2015). Next-generation sequencing for infectious disease diagnosis and management: a report of the association for molecular pathology. *J. Mol. Diagn.* 17 623–634. 10.1016/j.jmoldx.2015.07.004 26433313

[B95] León C. Ruiz-Santana S. Saavedra P. Castro C. Loza A. Zakariya I. (2016). Contribution of *Candida* biomarkers and DNA detection for the diagnosis of invasive candidiasis in ICU patients with severe abdominal conditions. *Crit. Care* 20:149. 10.1186/s13054-016-1324-3 PMC4867537 27181045

[B96] Liesman R. M. Strasburg A. P. Heitman A. K. Theel E. S. Patel R. Binnicker M. J. (2018). Evaluation of a commercial multiplex molecular panel for diagnosis of infectious meningitis and encephalitis. *J. Clin. Microbiol.* 56:e1927-17. 10.1128/JCM.01927-17 29436421 PMC5869843

[B97] Litvintseva A. P. Hurst S. Gade L. Frace M. A. Hilsabeck R. Schupp J. M. (2014). Whole-genome analysis of *Exserohilum rostratum* from an outbreak of fungal meningitis and other infections. *J. Clin. Microbiol.* 52 3216–3222. 10.1128/JCM.00936-14 24951807 PMC4313140

[B98] Lockhart S. R. Etienne K. A. Vallabhaneni S. Farooqi J. Chowdhary A. Govender N. P. (2017). Simultaneous emergence of multidrug-resistant Candida auris on 3 Continents confirmed by whole-genome sequencing and epidemiological analysis. *Clin. Infect. Dis.* 64 134–140. 10.1093/cid/ciw691 27988485 PMC5215215

[B99] Loeffler J. Mengoli C. Springer J. Bretagne S. Cuenca-Estrella M. Klingspor L. (2015). Analytical comparison of in vitro-spiked human serum and plasma for PCR-based detection of *Aspergillus fumigatus* DNA: a study by the European *Aspergillus* PCR initiative. *J. Clin. Microbiol.* 53 2838–2845. 10.1128/JCM.00906-15 26085614 PMC4540929

[B100] Lu Y. Ling G. Qiang C. Ming Q. Wu C. Wang K. (2011). PCR diagnosis of *Pneumocystis* pneumonia: a bivariate meta-analysis. *J. Clin. Microbiol.* 49 4361–4363. 10.1128/JCM.06066-11 22012008 PMC3232995

[B101] Macesic N. Morrissey C. O. Liew D. Bohensky M. A. Chen S. C. Gilroy N. M. (2017). Is a biomarker-based diagnostic strategy for invasive aspergillosis cost effective in high-risk haematology patients? *Med. Mycol*. 55 705–712. 10.1093/mmy/myw141 28131991

[B102] Maillet M. Maubon D. Brion J. P. François P. Molina L. Stahl J. P. (2014). *Pneumocystis jirovecii* (Pj) quantitative PCR to differentiate Pj pneumonia from Pj colonization in immunocompromised patients. *Eur. J. Clin. Microbiol. Infect. Dis.* 33 331–336. 10.1007/s10096-013-1960-3 23990137 PMC7101903

[B103] Malani A. N. Kauffman C. A. (2007). Changing epidemiology of rare mould infections: implications for therapy. *Drugs* 67 1803–1812. 10.2165/00003495-200767130-00001 17722951

[B104] Martel C. M. Parker J. E. Bader O. Weig M. Gross U. Warrilow A. G. (2010). Identification and characterisation of four azole-resistant erg3 mutants of *Candida albicans*. *Antimicrob. Agents Chemother.* 54 4527–4533. 10.1128/AAC.00348-10 20733039 PMC2976150

[B105] Maubon D. Dard C. Garnaud C. Cornet M. (2018). Profile of GenMark’s ePlex® blood culture identification fungal pathogen panel. *Expert Rev. Mol. Diagn.* 18 119–132. 10.1080/14737159.2018.1420476 29284316

[B106] McMullan R. Metwally L. Coyle P. V. Hedderwick S. McCloskey B. O’Neill H. J. (2008). A prospective clinical trial of a real-time polymerase chain reaction assay for the diagnosis of candidaemia in nonneutropenic, critically ill adults. *Clin. Infect. Dis.* 46 890–896. 10.1086/528690 18260751

[B107] McTaggart L. R. Copeland J. K. Surendra A. Wang P. W. Husain S. Coburn B. (2019). Mycobiome sequencing and analysis applied to fungal community profiling of the lower respiratory tract during fungal pathogenesis. *Front. Microbiol.* 10:512. 10.3389/fmicb.2019.00512 30930884 PMC6428700

[B108] Meletiadis J. Mavridou E. Melchers W. J. G. Mouton J. W. Verweij P. E. (2012). Epidemiological cutoff values for azoles and *Aspergillus fumigatus* based on a novel mathematical approach incorporating *cyp51A* sequence analysis. *Antimicrob. Agents Chemother.* 56 2524–2529. 10.1128/AAC.05959-11 22330922 PMC3346643

[B109] Mengoli C. Cruciani M. Barnes R. A. Loefffler J. Donnelly J. P. (2009). Use of PCR for diagnosis of invasive aspergillosis: systematic review and meta-analysis. *Lancet Infect. Dis.* 9 89–96. 10.1016/S1473-3099(09)70019-2 19179225

[B110] Messacar K. Parker S. K. Todd J. K. Dominguez S. R. (2017). Implementation of rapid molecular infectious disease diagnostics: the role of diagnostic and antimicrobial stewardship. *J. Clin. Microbiol.* 55 715–723. 10.1128/JCM.02264-16 28031432 PMC5328439

[B111] Meyer W. Irinyi L. Hoang M. T. V. Robert V. Garcia-Hermoso D. Desnos-Olivier M. (2019). Database establishment for the secondary fungal DNA barcode translational elongation factor 1alpha (TEF1alpha). *Genome Can.* 62 160–169. 10.1139/gen-2018-0083 30465691

[B112] Millon L. Herbrecht R. Grenouillet F. Morio F. Alanio A. Letscher-Bru V. (2015). Early diagnosis and monitoring of mucormycosis by detection of circulating DNA in serum: retrospective analysis of 44 cases collected through the French surveillance network of invasive fungal infections (RESSIF). *Clin. Microbiol. Infect.* 22 810.e1–810.e8. 10.1016/j.cmi.2015.12.006 26706615

[B113] Millon L. Larosa F. Lepiller Q. Legrand F. Rocchi S. Daquindau E. (2013). Quantitative polymerase chain reaction detection of circulating DNA in serum for early diagnosis of mucormycosis in immunocompromised patients. *Clin. Infect. Dis.* 56 e95–e101. 10.1093/cid/cit094 23420816

[B114] Millon L. Scherer E. Rocchi S. Bellanger A.-P. (2019). Molecular strategies to diagnose mucormycosis. *J. Fungi* 5:24. 10.3390/jof5010024 30897709 PMC6463105

[B115] Montesinos I. Delforge M. L. Ajjaham F. Brancart F. Hites M. Jacobs F. (2017). Evaluation of a new commercial real-time PCR assay for diagnosis of *Pneumocystis jirovecii* pneumonia and identification of dihydropteroate synthase (DHPS) mutations. *Diagn. Microbiol. Infect. Dis.* 87 32–36. 10.1016/j.diagmicrobio.2016.10.005 27789058

[B116] Morschhäuser J. Barker K. S. Liu T. T. Blaß-Warmuth J. Homayouni R. Rogers P. D. (2007). The transcription factor Mrr1p controls expression of the MDR1 efflux pump and mediates multidrug resistance in *Candida albicans*. *PLoS Pathog.* 3:e164. 10.1371/journal.ppat.0030164 17983269 PMC2048531

[B117] Morton C. O. White P. L. Barnes R. A. Klingspor L. Cuenca-Estrella M. Lagrou K. (2017). Determining the analytical specificity of PCR-based assays for the diagnosis of IA: what is *Aspergillus*? *Med. Mycol*. 55 402–403. 10.1093/mmy/myw093 28339744

[B118] Mulcahy-O’Grady H. Workentine M. L. (2016). The challenge and potential of metagenomics in the clinic. *Front. Immunol.* 7:29. 10.3389/fimmu.2016.00029 26870044 PMC4737888

[B119] Mylonakis E. Clancy C. J. Ostrosky-Zeichner L. Garey K. W. Alangaden G. J. Vazquez J. A. (2015). T2 magnetic resonance assay for the rapid diagnosis of candidemia in whole blood: a clinical trial. *Clin. Infect. Dis.* 60 892–899. 10.1093/cid/ciu959 25586686

[B120] Navalkele B. D. Revankar S. Chandrasekar P. (2017). *Candida auris*: a worrisome, globally emerging pathogen. *Expert Rev. Anti. Infect. Ther.* 15 819–827. 10.1080/14787210.20171364992 28783385

[B121] Neely L. A. Audeh M. Phung N. A. Min M. Suchocki A. Plourde D. (2013). T2 magnetic resonance enables nanoparticle-mediated rapid detection of candidemia in whole blood. *Sci. Transl. Med.* 5:182ra54. 10.1126/scitranslmed.3005377 23616121

[B122] Nguyen M. H. Wissel M. C. Shields R. K. Salomoni M. A. Hao B. Press E. G. (2012). Performance of Candida real-time polymerase chain reaction, β-D-glucan assay, and blood cultures in the diagnosis of invasive candidiasis. *Clin. Infect. Dis.* 54 1240–1248. 10.1128/JCM.00496-17 22431804

[B123] Nicholls S. M. Quick J. C. Tang S. Loman N. J. (2019). Ultra-deep, long-read nanopore sequencing of mock microbial community standards. *Gigascience* 8:giz043. 10.1093/gigascience/giz043 31089679 PMC6520541

[B124] Pagès A. Iriart X. Molinier L. Georges B. Berry A. Massip P. (2017). Cost effectiveness of I polymerase chain reaction detection and empirical antifungal treatment among patients with suspected fungal peritonitis in the intensive care unit. *Value Health* 20 1319–1328. 10.1016/j.jval.2017.06.009 29241891

[B125] Pasqualotto A. C. Falci D. R. (2016). Has *Aspergillus* PCR come to the age of maturity? *Mycopathol* 181 623–624. 10.1007/s11046-016-0033-4 27358190

[B126] Patch M. E. Weisz E. Cubillos A. Estrada S. J. Pfaller M. A. (2018). Impact of rapid, culture independent diagnosis of candidaemia and invasive candidiasis in a community health system. *J. Antimicrob. Chemother.* 73(Suppl. 4), iv27–iv30. 10.1093/jac/dky046 29608750

[B127] Perlin D. S. (2009). Antifungal drug resistance: do molecular methods provide a way forward? *Curr. Opin. Infect. Dis*. 22 568–573. 10.1097/QCO.0b013e3283321ce5 19741524 PMC3913535

[B128] Perlin D. S. (2015). Echinocandin resistance in *Candida*. *Clin. Infect. Dis.* 61 S612–S617. 10.1093/cid/civ791 26567278 PMC4643482

[B129] Perlin D. S. Wiederhold N. P. (2017). Culture-independent molecular methods for detection of antifungal resistance mechanisms and fungal identification. *J. Infect. Dis.* 216(Suppl. 3), S458–S465. 10.1093/infdis/jix121 28911041

[B130] Pfaller M. A. Castanheira M. (2016). Nosocomial candidiasis: antifungal stewardship and the importance of rapid diagnosis. *Med. Mycol.* 54 1–22. 10.1093/mmy/myv076 26385381

[B131] Pfaller M. A. Castanheira M. Lockhart S. R. Ahlquist A. M. Messer S. A. Jones R. N. (2012). Frequency of decreased susceptibility and resistance to echinocandins among fluconazole-resistant bloodstream isolates of *Candida glabrata*. *J. Clin. Microbiol.* 50 1199–1203. 10.1128/JCM.06112-11 22278842 PMC3318516

[B132] Pfeiffer C. D. Samsa G. P. Schell W. A. Reller L. B. Perfect J. R. Alexander B. D. (2011). Quantitation of Candida CFU in initial positive blood cultures. *J. Clin. Microbiol.* 49:2879. 10.1128/JCM.00609-11 21677065 PMC3147732

[B133] Pham C. D. Iqbal N. Bolden C. B. Kuykendall R. J. Harrison L. H. Farley M. M. (2014). Role of *FKS* mutations in *Candida glabrata*: MIC values, echinocandin resistance, and multidrug reisstance. *Antimicrob. Agents Chemother.* 58 4690–4696. 10.1128/AAC.03255-14 24890592 PMC4136002

[B134] Rahn S. Schuck A. Kondakci M. Haas R. Neuhausen N. Pfeffer K. (2016). A novel comprehensive set of fungal real time PCR (fuPCR) for the detection of fungi in immunocompromised haematological patients – a pilot study. *Int. J. Med. Microbiol.* 306 611–623. 10.1016/j.ijmm.2016.10.003 27765533

[B135] Rath P.-M. Steinmann J. (2018). Overview of commercially available PCR assays for the detection of *Aspergillus* spp. DNA in patient samples. *Front. Microbiol.* 9:740. 10.3389/fmicb.2018.00740 29740403 PMC5928133

[B136] Rickerts V. (2016). Identification of fungal pathogens in formalin-fixed, paraffin-embedded tissue samples by molecular methods. *Fungal Biol.* 120 279–287. 10.1016/j.funbio.2015.07.002 26781382

[B137] Rickerts V. Khot P. D. Myerson D. Ko D. L. Lambrecht E. Fredricks D. N. (2011). Comparison of quantitative real time PCR with Sequencing and ribosomal RNA-FISH for the identification of fungi in formalin fixed, paraffin-embedded tissue specimens. *BMC Infect. Dis.* 11:202. 10.1186/1471-2334-11-202 21791040 PMC3160998

[B138] Sabino R. Simões H. Verissimo C. (2019). Detection of deep fungal infections: a polyphasic approach. *J. Med. Microbiol.* 68 81–86. 10.1099/jmm.0.000883 30480509

[B139] Salter S. J. Cox M. J. Turek E. M. Calus S. T. Cookson W. O. Moffatt M. F. (2014). Reagent and laboratory contamination can critically impact sequence-based microbiome analyses. *BMC Biol.* 12:87. 10.1186/s12915-014-0087-z 25387460 PMC4228153

[B140] Sanderson N. D. Street T. L. Foster D. Swann J. Atkins B. L. Brent A. J. (2018). Real-time analysis of nanopore-based metagenomic sequencing from infected orthopaedic devices. *BMC Genomics* 19:714. 10.1186/s12864-018-5094-y 30261842 PMC6161345

[B141] Sasso M. Chastang-Dumas E. Bastide S. Alonso S. Lechiche C. Bourgeois N. (2016). Performance of four real-time PCR assays for diagnosis of *Pneumocystis jirovecii* pneumonia. *J. Clin. Microbiol.* 54 625–630. 10.1128/JCM.02876-15 26719435 PMC4767985

[B142] Scherer E. Iriart X. Bellanger A. P. Dupont D. Guitard J. Gabriel F. (2018). Quantitative PCR (qPCR) detection of Mucorales DNA in bronchoalveolar lavage fluid to diagnose pulmonary mucormycosis. *J. Clin. Microbiol.* 56:e00289-18. 10.1128/JCM.00289-18 29875192 PMC6062785

[B143] Schirmer M. Ijaz U. Z. D’Amore R. Hall N. Sloan W. T. Quince C. (2015). Insight into biases and sequencing errors for amplicon sequencing with the Illumina MiSeq platform. *Nucleic Acids Res.* 43:e37. 10.1093/nar/gku1341 25586220 PMC4381044

[B144] Schloss P. D. Westcott S. L. (2011). Assessing and improving methods used in operational taxonomic unit-based approaches for 16S rRNA gene sequence analysis. *Appl. Environ. Microbiol.* 77 3219–3226. 10.1128/AEM.02810-10 21421784 PMC3126452

[B145] Schoch C. L. Robbertse B. Robert V. Vu D. Cardinali G. Irinyi L. (2014). Finding needles in haystacks: linking scientific names, reference specimens and molecular data for Fungi. *Database* 2014:bau061. 10.1093/database/bau061 24980130 PMC4075928

[B146] Schoch C. L. Seifert K. A. Huhndorf S. Robert V. Spouge J. L. Levesque C. A. (2012). Nuclear ribosomal internal transcribed spacer (ITS) region as a universal DNA barcode marker for Fungi. *Proc. Natl. Acad. Sci. U.S.A.* 109 6241–6246. 10.1073/pnas.1117018109 22454494 PMC3341068

[B147] Sexton A. J. Bentz M. L. Welsh R. M. Litvintseva A. P. (2018). Evaluation of a new T2 Magnetic Resonance assay for rapid detection of emergent fungal pathogen *Candida auris* on clinical skin swab samples. *Mycoses* 61 786–790. 10.1111/myc.12817 29938838

[B148] Sherry L. Ramage G. Kean R. Borman A. Johnson E. M. Richardson M. D. (2017). Biofilm-forming capability of highly virulent, multidrug resistant *Candida auris*. *Emerg. Infect. Dis.* 23 328–331. 10.3201/eid2302.161320 28098553 PMC5324806

[B149] Shields R. K. Nguyen M. H. Press E. G. Kwa A. L. Cheng S. Du C. (2012). The presence of an *FKS* mutation rather than MIC is an independent risk factor for failure of echinocandin therapy among patients with invasive candidiasis due to *Candida glabrata*. *Antimicrob. Agents Chemother.* 56 4862–4869. 10.1128/AAC.00027-12 22751546 PMC3421882

[B150] Snelders E. Huis In ‘t Veld R. A. Rijs A. J. Kema G. H. Melchers W. J. Verweij P. E. (2009). Possible environmental origin of resistance of *Aspergillus fumigatus* to medical triazoles. *Appl. Environ. Microbiol*. 75 4053–4057. 10.1128/AEM.00231-09 19376899 PMC2698372

[B151] Springer J. Goldenberger D. Schmidt F. Weisser M. Wehrle-Wieland E. Einsele H. (2016a). Development and application of two independent real-time PCR assays to detect clinically relevant Mucorales species. *J. Med. Microbiol.* 65 227–234. 10.1099/jmm.0.000218 26743820

[B152] Springer J. Lackner M. Ensinger C. Risslegger B. Morton C. O. Nachbaur D. (2016b). Clinical evaluation of a Mucorales-specific real-time PCR assay in tissue and serum samples. *J. Med. Microbiol.* 65 1414–1421. 10.1099/jmm.0.000375 27902424

[B153] Stielow J. B. Lévesque C. A. Seifert K. A. Meyer W. Iriny L. Smits D. (2015). One fungus, which genes? Development and assessment of universal primers for potential secondary fungal DNA barcodes. *Persoonia* 35 242–263. 10.3767/003158515X689135 26823635 PMC4713107

[B154] Strong M. J. Xu G. Morici L. Splinter Bon-Durant S. Baddoo M. Lin Z. (2014). Microbial contamination in next generation sequencing: implications for sequence-based analysis of clinical samples. *PLoS Pathog.* 10:e1004437. 10.1371/journal.ppat.1004437 25412476 PMC4239086

[B155] Sugawara Y. Nakase K. Nakamurra A. Ohishi K. Sugimoto Y. Fujieda A. (2013). Clinical utility of a panfungal polymerase chain reaction assay for invasive fungal diseases in patients with haematologic disorders. *Eur. J. Haematol.* 90 331–339. 10.1111/ejh.12078 23360173

[B156] Summah H. Zhu Y.-G. Falagas M. E. Vouloumanou E. K. Qu J. M. (2013). Use of real-time polymerase chain reaction for the diagnosis of *Pneumocystis* pneumonia in immunocompromised patients: a meta-analysis. *Chin. Med. J.* 126 1965–1973. 23673119

[B157] Sun W. Wang K. Gao W. Su Z. Qian Q. Lu X. (2011). Evaluation of PCR on bronchoalveolar lavagel fluid for the diagnosis of invasive aspergillosis: a bivariate meta-analysis and systematic review. *PLoS One* 6:228467. 10.1371/journal.pone.0028467 22164295 PMC3229594

[B158] Talbot J. J. Subedi S. Halliday C. L. Hibbs D. E. Lai F. Lopez-Ruiz F. J. (2018). Surveillance for azole resistance in clinical and environmental isolates of *Aspergillus fumigatus* in Australia and cyp51A homology modelling of azole-resistant isolates. *J. Antimicrob. Chemother.* 73 2347–2351. 10.1093/jac/dky187 29846581

[B159] Trubiano J. A. Dennison A. M. Morrissey C. O. Chua K. Y. Halliday C. L. Chen S. C. (2016). Clinical utility of panfungal polymerase chain reaction for the diagnosis of invasive fungal disease: a single centre experience. *Med. Mycol.* 54 138–146. 10.1093/mmy/myv092 26527638

[B160] Tyler A. D. Mataseje L. Urfano C. J. Schmidt L. Antonation K. S. Mulvey M. R. (2018). Evaluation of Oxford Nanopore’s MinION sequencing device for microbial whole genome sequencing applications. *Sci. Rep.* 8:10931. 10.1038/s41598-018-29334-5 PMC6053456 30026559

[B161] Ullmann A. J. Aguado J. M. Arikan-Adkagli S. Denning D. Q. Groll A. H. Lagrou K. (2018). Diagnosis and management of *Aspergillus* diseases: executive summary of the 2017 ESCMID-ECMM-ERS guideline. *Clin. Microbiol. Infect.* 24 e1–e38. 10.1016/j.cmi.2018.01.002 29544767

[B162] Valero C. de la Cruz-Villar L. Zaragoza O. Buitrago M. J. (2016). New panfungal real-time PCR assay for diagnosis of invasive fungal infections. *J. Clin. Microbiol.* 54 2910–2918. 10.1128/jcm.01580-16 27629898 PMC5121379

[B163] Vallabhaneni S. Benedict K. Derado G. Mody R. K. (2017). Trends in hospitalizations related to invasive aspergillosis and mucormycosis in the United States, 2000-2013. *Open Forum Infect. Dis.* 4:ofw268. 10.1093/ofid/ofw268 28480260 PMC5413990

[B164] van der Linden J. W. Camps S. M. Kampinga G. A. Arends J. P. Debets-Ossenkopp Y. J. Haas P. J. (2013). Aspergillosis due to voriconazole highly resistant *Aspergillus fumgiatus* and recovery of genetially related isolates from domiciles. *Clin. Infect. Dis.* 57 513–520. 10.1093/cid/cit320 23667263

[B165] Vermitsky J. P. Learhart K. D. Smith W. L. Homayouni R. Edlind T. D. Rogers P. D. (2006). Pdr1 regulates multidrug resistance in *Candida glabrata*: gene disruption and genome-wide expression studies. *Mol. Microbiol.* 61 704–722. 10.1111/j.1365-2958.2006.05235.x 16803598

[B166] Walker B. Powers-Fletcher M. V. Schmidt R. L. Hanson K. E. (2016). Cost-effectiveness analysis of multiplex PCR with magnetic resonance detection versus empiric or blood culture-directed therapy for management of suspected candidaemia. *J. Clin. Microbiol.* 54 718–726. 10.1128/JCM.02971-15 26739159 PMC4767978

[B167] Wehrle-Wieland E. Affolter K. Goldenberger D. Tschudin Sutter S. Halter J. Passweg J. (2018). Diagnosis of invasive mold diseases in patients with hematological malignancies using *Aspergillus*, Mucorales, and panfungal PCR in BAL. *Transpl. Infect. Dis.* 20:e12953. 10.1111/tid.12953 29896857

[B168] White P. L. (2019). Recent advances and novel approaches in laboratory-based diagnostic mycology. *Med. Mycol.* 57 S259–S266. 10.1093/mmy/myy159 31292661

[B169] White P. L. Backx M. Barnes R. A. (2017a). Diagnosis and management of *Pneumocystis jirovecii* infection. *Expert Rev. Anti. Infect. Ther.* 15 435–447. 10.1080/14787210.2017.1305887 28287010

[B170] White P. L. Barnes R. A. Springer J. Klingspor L. Cuenca-Estrella M. Morton C. O. (2015). Clinical performance of *Aspergillus* PCR for testing serum and plasma: a study by the European *Aspergillus* PCR Initiative. *J. Clin. Microbiol.* 53 2832–2837. 10.1128/JCM.00905-15 26085618 PMC4540904

[B171] White P. L. Bretagne S. Klingspor L. Melchers W. J. McCulloch E. Schulz B. (2010a). *Aspergillus* PCR: one step closer to standaridization. *J. Clin. Microbiol.* 48 1231–1240. 10.1128/JCM.01767-09 20147637 PMC2849576

[B172] White P. L. Perry M. D. Loeffler J. Melchers W. Klingspor L. Bretagne S. (2010b). Critical stages of extracting DNA from *Aspergillus fumigatus* in whole-blood specimens. *J. Clin. Microbiol.* 48 3753–3755. 10.1128/JCM.01466-10 20720026 PMC2953093

[B173] White P. L. Mengoli C. Bretagne S. Cuenca-Estrella M. Finnstrom N. Klingspor L. (2011). Evaluation of *Aspergillus* PCR protocols for testing serum specimens. *J. Clin. Microbiol.* 49 3842–3848. 10.1128/JCM.05316-11 21940479 PMC3209112

[B174] White P. L. Posso R. B. Barnes R. A. (2017b). An analytical and clinical evaluation of the PathoNostics AsperGenius® Assay for the detection of invasive aspergillosis and resistance to azole antifungal drugs direct from plasma samples. *J. Clin. Microbiol.* 55 2356–2366. 10.1128/jcm.00411-17 28515217 PMC5527413

[B175] White T. J. Bruns T. Lee S. Taylor J. (1990). “Amplification and direct sequencing of fungal ribosomal RNA Genes for phylogenetics,” in *PCR Protocols: A Guide to Methods and Applications*, eds Innis M. A. Gelfand D. H. Sninsky J. J. White T. J. (New York, NY: Academic Press), 315–322. 10.1016/b978-0-12-372180-8.50042-1

[B176] Zacharioudakis I. M. Zervou F. N. Mylonakis E. (2018). T2 magnetic resonance assay: overview of available data and clinical implications. *J. Fungi* 4:45. 10.3390/jof4020045 29617284 PMC6023470

[B177] Zeller I. Schabereiter-Gurtner C. Mihalits V. Selitsch B. Barousch W. Hirschi A. M. (2017). Detection of fungal pathogens by a new broad range real-time PCR assay targeting the fungal ITS2 region. *J. Med. Microbiol.* 66 1383–1392. 10.1099/jmm.0.000575 28884671

[B178] Zhang S. X. (2013). Enhancing molecular approaches for diagnosis of fungal infections. *Future Microbiol.* 8 1599–1611. 10.2217/fmb.13.120 24266359

[B179] Zhao Y. Nagasaki Y. Kordalewska M. Press E. G. Shields R. K. Nguyen M. H. (2016). Rapid detection of of *FKS*-associated echinocandin resistance in *Candida glabrata*. *Antimicrob. Agents Chemother.* 60 6573–6577. 10.1128/AAC.01574-16 27550360 PMC5075061

[B180] Zhou Y. Wylie K. M. El Feghaly R. E. Mihindukulasuriya K. A. Elward A. Haslam D. B. (2016). Metagenomic approach for identification of the pathogens associated with diarrhea in stool specimens. *J. Clin. Microbiol.* 54 368–375. 10.1128/JCM.01965-15 26637379 PMC4733167

